# Imidazole and Benzimidazole Modified Half-Sandwich Iridium^III^ *N*-Heterocyclic Carbene Complexes: Synthesis, Anticancer Application, and Organelle Targeting

**DOI:** 10.3389/fchem.2020.00182

**Published:** 2020-03-17

**Authors:** Xicheng Liu, Yali Han, Xingxing Ge, Zhe Liu

**Affiliations:** The Key Laboratory of Life-Organic Analysis, Key Laboratory of Pharmaceutical Intermediates and Analysis of Natural Medicine, School of Chemistry and Chemical Engineering, Institute of Anticancer Agents Development and Theranostic Application, Qufu Normal University, Qufu, China

**Keywords:** iridium(III) compounds, anticancer, *N*-heterocyclic carbene, organelle targeting, imidazole

## Abstract

Herein, we report the synthesis, characterization and anticancer activity of a series of half-sandwich iridium^III^ imidazole and benzimidazole *N*-heterocyclic carbene (NHC) anticancer complexes, and the general formula of which can be expressed as [(η^5^-Cp^x^)Ir(C^∧^N)Cl]Cl (Cp^x^: pentamethylcyclopentadienyl (Cp^*^) or biphenyl derivatives (Cp^xbiph^); C^∧^N: imidazole and benzimidazole NHC chelating ligands). Compared with *cis*-platin, these complexes showed interesting antitumor activity against A549 cells. Complexes could bind to bovine serum albumin (BSA) by means of static quenching mode, catalyze the oxidation of nicotinamide adenine dinucleotide (NADH) and increase the levels of reactive oxygen species (ROS). Meanwhile, these complexes could arrest the cell cycles of A549 cells and influence the mitochondrial membrane potential significantly. Due to the inherent luminescence property, laser confocal test show that complexes could enter cells followed an energy-dependent mechanism and effectively accumulate in lysosome (the value of Pearson's co-localization coefficient is 0.70 after 1 h), further destroy lysosome integrity and induce apoptosis.

## Introduction

When cell growth is uncontrollable and abnormal proliferation occurs, malignant tumors are formed (Valastyan and Weinberg Robert, 2011; Grossi et al., 2016). Most forms of disseminated tumors are currently untreatable, although developments in chemotherapy over the past years have led to the treatment of various cancers (Thota et al., 2018). *Cis*-platin exerts good and broad-spectrum anticancer effects (Deubel and Lau, 2006; Romero-Canelón and Sadler, 2013; Muhammad and Guo, 2014), but, when used extensively, platinum-based drugs may promote drug resistance and instability; become insoluble in biological media; and become toxic to blood and gastrointestinal tract (Ma et al., 2014; Liu et al., 2015). Thus, various organometallic anticancer drugs, such as tin, iridium, ruthenium, and osmium anticancer complexes, have been developed (Sun and Che, 2009; Lu et al., 2015; Zeng et al., 2017; Ge et al., 2019; Li et al., 2019).

Ir^III^ complexes are inert, half-sandwich Ir^III^ complexes (a type of [(Cp^x^)Ir(L^∧^L')Z]X) show good anticancer activities (Richens, 2005), especially for tumors resistant to platinum-based drugs (Singh et al., 2010). These complexes are composed of cyclopentadienyl ligands (Cp^x^), chelating ligands (L^∧^L'), leaving groups (Z), and counter ions (X), each of which has an inherent effect on the anticancer activity of these complexes (Liu et al., 2011a). The types and positions of substituents on L^∧^L'-chelating ligands are the most studied and finely regulated for the effective modulation of targeted sites, the lipid solubility and even the anticancer activities of these complexes (Yellol et al., 2013; Zamora et al., 2015; Hao et al., 2019). The anticancer activity of a simple half-sandwich Ir^III^ complex increases nearly 10 times when the bipyridine chelating ligand (N^∧^N) is replaced by a phenyl pyridine ligand (C^∧^N) (Liu and Sadler, 2014). The IC_50_ value (the concentration at which the growth of 50% of the cells is inhibited) of Ir^III^ complex can be reduced by nearly 100 times by controlling the binding mode of bipyridine chelate ligand substituents and the number of triphenylamine (TPA) units (He et al., 2019).

**Figure 1:**
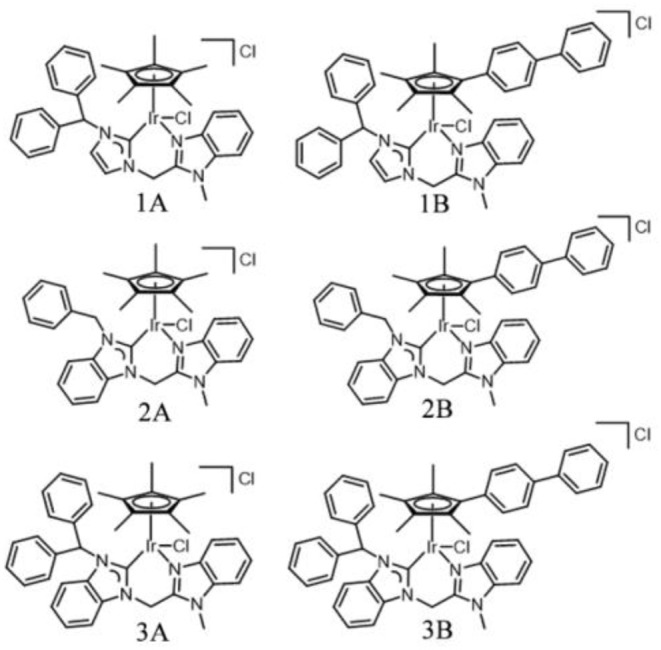
Ir^III^ NHC complexes **1A**–**3B** studied in this work.

As a chelating ligand, *N*-heterocyclic carbene (NHC) provides an *s*-donor, which not only ensures the high stability of a metal complex, but also prevents its hydrolyzation in various physiological media. Transition metal complexes containing NHC have been widely used in the field of catalysis (Powell et al., 2010; Schuh et al., 2012; Pellei et al., 2015). These complexes affect different catalytic modes and even change the progress of the cyclic metal complex catalytic process (Zou et al., 2018). Metal iridium complexes containing NHC targets lysosomes and mitochondria selectively, induces lysosome damage, alters mitochondrial membrane potential, blocks the cell growth cycle, and promotes apoptosis (Han et al., 2018). In the present study, a series of half-sandwich Ir^III^ NHC complexes were synthesized and characterized in detail, and NHC pro-ligands were coordinated with metal iridium in the mode of C^∧^N (Figure 1). MTT assay was used to assess the anticancer activity of Ir^III^ NHC complexes in A549 cells lines. The anticancer mechanism of these complexes were also studied by flow cytometer and laser scanning confocal microscope. The results indicated that half-sandwich Ir^III^ NHC complexes may be promising candidate for anticancer drugs and broaden the research scope in this field.

## Results and Discussion

### Synthesis and Characterization

**Scheme 1:**
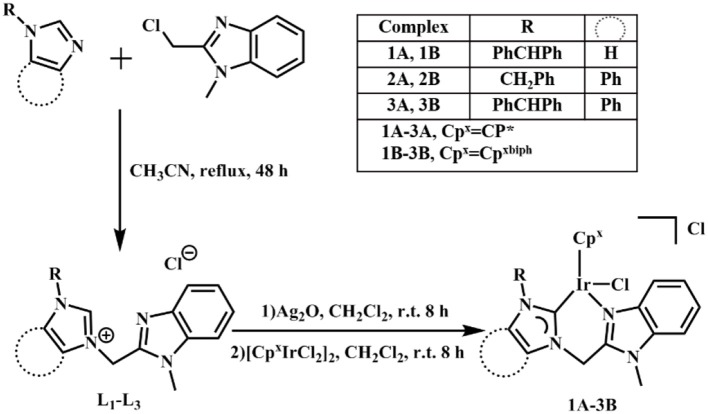
Synthesis of chelating ligands **L**_**1**_–**L**_**3**_ and Ir^III^ NHC complexes **1A**–**3B**.

NHC pro-ligands and a series of half-sandwich Ir^III^ NHC complexes of the type [(η^5^-Cp^x^)Ir(C^∧^N)Cl]Cl (**1A**-**3B**) containing Cp ring and its biphenyl derivative (Scheme 1), were synthesized and characterized for the first time. Then, 1-diphenylmethylimidazole, 1-benzylbenzimidazole and 1-benzhydrylbenzi- midazole were synthesized with imidazole and benzimidazole as raw materials and then reacted with 2-(chloromethyl)-1-methylbenzimidazole (Figure S1). The products were named **L**_**1**_, **L**_**2**_, and **L**_**3**_, respectively. The target complexes were obtained by catalyzing the reaction between the chelating ligands (**L**_**1**_–**L**_**3**_) and dimers (Cp^*^ and Cp^xbiph^) in dichloromethane with the use of silver oxide (Ag_2_O) at ambient temperature and good yields of 59–69%. The target complexes were isolated as Cl salt and characterized by ^1^H NMR (Figure S2), ^13^C NMR (Figure S3), mass spectroscopy (Figure S4) and elemental analysis. The ^1^H NMR spectra and MS spectral data are provided in the support information, as shown in Tables S1, S2. At the same time, we characterized the carbon in the complex, in which the peak shift of *C*-Ir is between 148.66 and 167.58 ppm, and the peak shift of N*C*N is between 138.56 and 150.24 ppm. After simple purification, the complexes were isolated as powdered and non-hygroscopic solids. The complexes were highly soluble in dichloromethane, chloroform, and dimethyl sulfoxide; partially soluble in methanol; and insoluble in ether, petroleum ether, and *n*-hexane.

M-OH_2_ complexes usually have higher anticancer activities than M-Cl complexes (Liu et al., 2011b). Hydrolysis behavior of complex **3B** in 50% CD_3_OD-*d*_4_/50% D_2_O (*v/v*) and 50% MeOH/50% H_2_O (*v/v*) was monitored by ^1^H NMR (Figure S5) and ultraviolet-visible spectrophotometry (UV-Vis; Figure S6) at 298 K, respectively. No obvious changes were observed in the ^1^H NMR and UV-Vis spectra, which indicating that Ir^III^ NHC complexes did not hydrolyze and the structure was stable high water content. Thus, the Ir^III^ NHC complexes are sufficiently stable for the bioassay.

### Cytotoxicity Test

**Figure 2:**
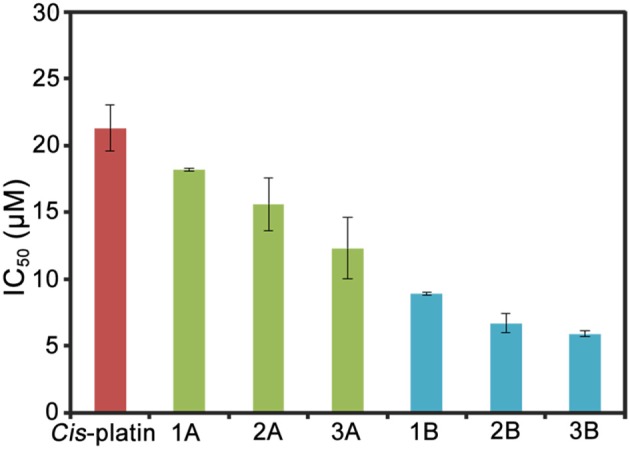
Histograms of IC_50_ values for complexes **1A**–**3B** and *cis*-platin against A549 cells after 24 h.

Lung cancer has a high mortality rate in developed and developing countries, therefore, A549 cells were selected as the model of biological research (Jemal et al., 2011). The antiproliferative activities of half-sandwich Ir^III^ complexes [(Cp^x^)Ir(L^∧^L')Z]Cl are affected by changes in cyclopentadienyl or chelating ligand structures of the complexes (Liu and Sadler, 2014). The antiproliferative activities of half-sandwich Ir^III^ complexes **1A**–**3B** against A549 cancer cells after 24 h are shown in Table 1 and Figure 2. These complexes exhibit better activity than imidazolium salts (**L1–L3**) and [Cp^x^IrCl_2_]_2_ (IC_50_: > 100), meanwhile, **1A**–**3B** had higher IC_50_ values than *cis*-platin (5.9–18.2 μM), especially complex **3B** (3.6 times of *cis*-platin).

**Table 1:** IC_50_ values of complexes **1A**–**3B** and *cis*-platin against A549 cancer cells after 24 h exposure.

**Complexes**	**IC_**50**_ (μM)**
[(η^5^-C_5_Me_5_)Ir(**L**_**1**_)Cl]Cl (**1A**)	18.2 ± 0.1
[(η^5^-C_5_Me_5_)Ir(**L**_**2**_)Cl]Cl (**2A**)	15.6 ± 2.0
[(η^5^-C_5_Me_5_)Ir(**L**_**3**_)Cl]Cl (**3A**)	12.3 ± 2.3
[(η^5^-C_5_Me_4_C_6_H_4_C_6_H_5_)Ir(**L**_**1**_)Cl]Cl (**1B**)	8.9 ± 0.1
[(η^5^-C_5_Me_4_C_6_H_4_C_6_H_5_)Ir(**L**_**2**_)Cl]Cl (**2B**)	6.7 ± 0.7
[(η^5^-C_5_Me_4_C_6_H_4_C_6_H_5_)Ir(**L**_**3**_)Cl]Cl (**3B**)	5.9 ± 0.2
*Cis*-platin	21.3 ± 1.7

This result is consistent with the previous study, which showed that a Cp^xbiph^ group with strong electron donor capacity effectively improved the anticancer potential of Ir^III^ complexes (Wang et al., 2017). **2A** and **2B** formed large conjugated systems with strong electron donor capacities after benzene rings were introduced to their imidazole groups and thus showed better anticancer activity than **1A** and **1B**, where benzene rings were on the terminal methyl groups **3A** and **3B** showed the best anticancer potential because large organic ligands, which effectively enhanced the lipophilicity of the complexes. **2A**, **2B**, and **3B** had logP values (oil/water partition coefficient) were 0.92, 1.37, and 1.56, respectively, which further confirmed the sizes of the hydrophilic and lipophilic Ir^III^ NHC complexes (Zhang et al., 2018).

### Protein Bingding Assay

**Figure 3:**
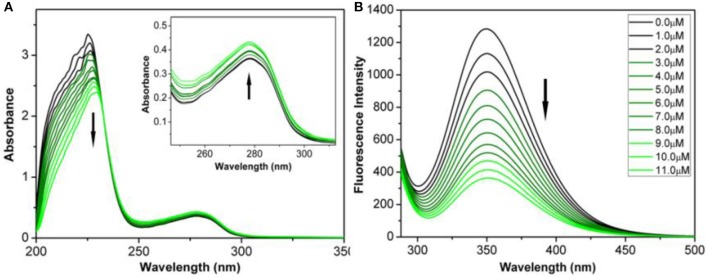
**(A)** UV-Vis spectrum of BSA reacted with complex **3B** (0–11 μM) in Tris-HCl/NaCl buffer solution of pH=7.2. Inset: Wavelength range: 250–300 nm. **(B)** Fluorescence spectra (λ_ex_ = 280 nm; λ_em_ = 343 nm) of complex **3B** (0–11 μM) reacted with BSA. Arrows: The change tendency of UV-Vis spectrum and fluorescence intensity with the addition of complex **3B**.

Serum albumin (SA) has significant binding properties and is an important drug delivery medium *in vivo* (Chen et al., 2016; Esteghamat-Panah et al., 2017). Bovine serum albumin (BSA) has the structural homology with human serum albumin (HSA), can be easily purified, and shows good stability and is thus an economical and effective protein binding model (Naz et al., 2017). The interactions of complexes with BSA were analyzed by using UV-Vis absorption and fluorescence spectrum (Figure 3).

As shown in Figure 3A and Figure S7A, the maximum absorption at 228 nm (the absorption of BSA) decreased when the concentrations of complexes **1B**–**3B** increased. The decrease indicated that the complexes combined with BSA through alpha-helical interference. The absorption peaks increased gradually at 278 nm. This result demonstrated that the complexes changed the microenvironments of the three aromatic acid residues (tryptophan, tyrosine and phenylalanine) in BSA (Zhang et al., 2015; Baral et al., 2017).

The molecular environment information near the fluorophore molecule was obtained by synchronous fluorescence spectroscopy at a low concentration (Jayabharathi et al., 2011). The use of Δλ = 15 nm and Δλ = 60 nm corresponds to the spectral characteristics of tyrosine residues and tryptophan residues, respectively. As shown in Figure S8, with the increase of the complexes, the synchronous fluorescence intensity decreased gradually. When Δλ = 15 nm, the emission wavelength red shifted to 1~3 nm (285 nm). Meanwhile, no change was observed when Δλ = 60 nm. Thus, tyrosine was more affected than tryptophan by the binding of Ir^III^ NHC complexes to BSA.

The properties of the bonds between the complexes and BSA were further examined by fluorescence emission spectrum. The fluorescence spectra were calibrated for “internal filter” effect correction (Pacheco and Bruzzone, 2013). As shown in Figure 3B and Figure S7B, the fluorescence intensity of BSA (~343 nm) was quenched obviously when the concentrations of complexes **1B–3B** increased. The possible quenching mechanism can be interpreted by using the Stern-Volmer equation and the Scatchard equation (Chatterjee and Mukherjee, 2014), *K*_*sv*_(Stern-Volmer quenching constant), *K*_*q*_(quenching rate constant), *K*_*b*_ (banding constant), and *n* (number of banding site) were then calculated. The *K*_*q*_ values of complexes **1B–3B** were 1.82 × 10^12^, 2.34 × 10^12^ and 2.66 × 10^12^ M^−1^ s^−1^ (Figure S9 and Table S3), respectively, which were about two orders of magnitude higher than the value of a pure dynamic quenching mechanism (2.0 × 10^10^ M^−1^ s^−1^). The results indicated that the half-sandwich Ir^III^ NHC complexes interacted with BSA in a static quenching mode. Additionally, **3B** has the largest *K*_*b*_ (1.13 × 10^−4^ M^−1^) and *n* (1.31), which were consistent with the results of MTT assay that complex **3B** has better best anticancer activity than **1B–3B**. The results indicate that Ir^III^ NHC complexes can be effectively combined with BSA, and thus, BSA can be considered as an excellent carrier for delivering these anticancer complexes *in vivo*.

### Apoptosis Assay

**Figure 4:**
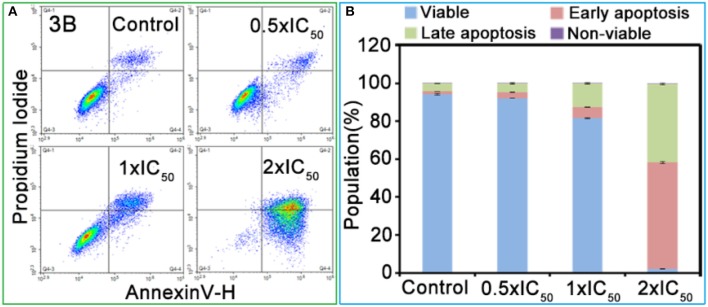
**(A)** Apoptosis analysis of A549 cancer cells after 24 h of exposure to complex **3B** at 310 K determined by flow cytometry using Annexin V-FITC*/*PI staining. **(B)** Histograms were the populations of A549 cells in four stages treated by complex **3B**. Data are quoted as mean ± SD of three replicates.

The effect of Ir^III^ complexes inducing apoptosis was determined by exposing the A549 cells to complex **3B** with a concentration of 0.5 × IC_50_, 1.0 × IC_50_ and 2.0 × IC_50_ for 24 h. The cells were stained with AnnexinV/ Propidium Iodide, then detected by flow cytometry (Rubbiani et al., 2011). As shown in Figure 4 and Table S4, complex **3B** induced apoptosis in a concentration-dependent manner, and this condition was mainly observed in early apoptosis and late apoptosis. At 2.0 × IC_50_, 97.6% (early apoptosis 56.2% + late apoptosis 41.4%) of the cells underwent apoptosis, and no considerable increase in increased necrotic population was detected. These results indicated that the Ir^III^ NHC complexes induced apoptosis effectively and exhibited favorable anticancer activity.

Reactive oxygen species (ROS) are mainly produced and stored by the mitochondria and play an important role in the regulation cell apoptosis and represent a pathway for oxidation anticancer mechanisms (Trachootham et al., 2009). After the 24 h treatment using complex **3B**, the ROS levels in the A549 cells changed (Figure S10). When the concentration of complex **3B** changed from 0.25 × IC_50_ to 0.5 × IC_50_, the ROS levels in the A549 cells were 1.3 and 1.6 times of the ROS level of the negative control (Table S5). This result is consistent with the result of the nicotinamide adenine dinucleotide (NADH) catalytic test. NADH (reduced coenzyme) can provide hydrides for Ir^III^ complexes and lead to the production of ROS, which providing a possibility anticancer mechanism of oxidation. To investigate the catalytic ability of complex for NADH, the reactions of complex **3B** (ca. 1 μM) with NADH (100 μM) in 60% MeOH/40% H_2_O (*v/v*) were monitored by UV-Vis spectrum at 298 K (Figure S11). The conversion of NADH to NAD^+^ was detected by measuring the changes at 339 and 259 nm (the absorption peak of NADH and NAD^+^, respectively). As shown, with the increase of the concentration of **3B**, there is a noticeable reduce and increase at 339 and 259 nm, respectively, which confirmed the catalytic activity of these complexes.

The relationship between anticancer mechanism and cell cycle arrest was determined by analyzing the cell cycle arrest of complex **3B** in A549 cells through flow cytometry. Compared with the untreated cells, the A549 cells were mainly arrested in the sub-G_1_ and G_2_/M, and the cell population increased from 52.0 to 66.8% and from 11.1 to 20.1%, respectively, when the concentration of complex **3B** increased from 0.25 × IC_50_ to 2.0 × IC_50_ (Figure S12 and Table S6). The result suggested that complex **3B** may blocked the synthesis of RNA, ribosomes, and several proteins (Senese et al., 2014). The 6.9% increase in the percentage of cells in the S phase indicated that complex **3B** inhibited the synthesis of DNA, histone, and some DNA replication-related enzymes and that Ir^III^ NHC complexes arrested tumor cell cycle at multiple stages and induced apoptosis (Sabharwal and Schumacker, 2014).

**Figure 5:**
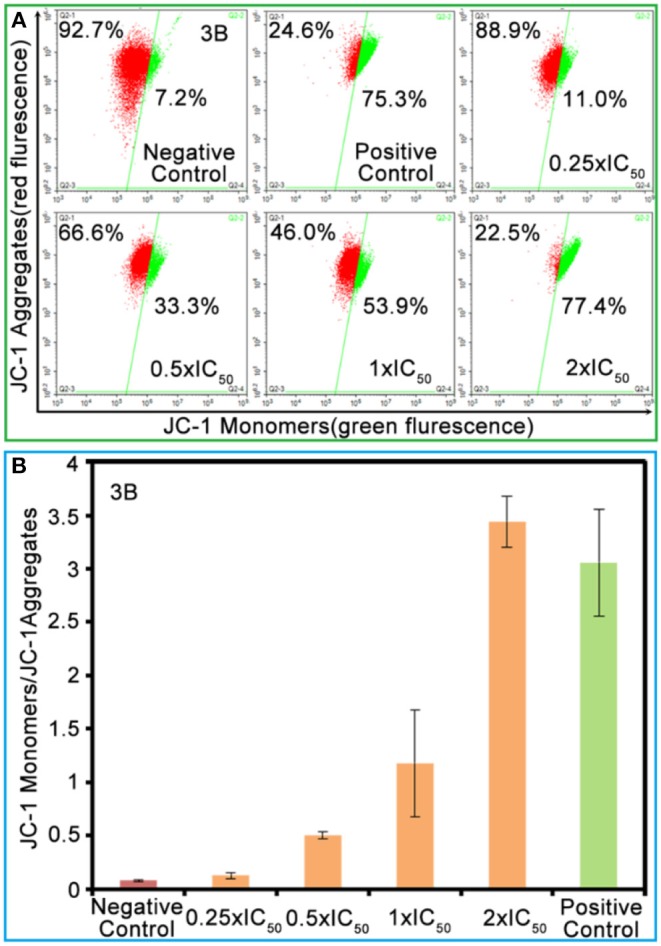
**(A)** The green/red fluorescence intensity ratio for JC-1 induced by complex **3B** at the concentrations of 0.25 × IC_50_, 0.5 × IC_50_, 1.0 × IC_50_, and 2.0 × IC_50_. **(B)** Histograms of JC-1 monomer (green)/JC-1 aggregates (red) at different concentrations of complex **3B**. Data were referenced as average ±SD. of three repeated tests.

The mitochondria are the main energy-producing sites in cells and play an essential role in apoptosis. Once the mitochondrial membrane is damaged, releases signals, and the damage leads to mitochondrial dysfunction and then induces apoptosis (Nichi et al., 2016). The degree of mitochondrial dysfunction can be evaluated by measuring the changes in mitochondrial membrane potential (MMP; Gómez-Sintes et al., 2016). The change in the green/red fluorescence intensity ratio for JC-1 (an ideal fluorescent probe widely used for detecting MMP) reflects the depolarization of MMP. As shown in Figure 5 and Table S7, the population of mitochondrial membrane depolarized cells changed from 11.0 to 77.4% with the increase of complex **3B** (from 0.25 × IC_50_ to 2.0 × IC_50_). Increase in the ratio of JC-1 green/red fluorescence intensity (Figure 5B) confirmed that the Ir^III^ NHC complexes affect the integrity of the mitochondrial membrane and induce apoptosis.

### Cellular Localization and Uptake Mechanism

**Figure 6:**
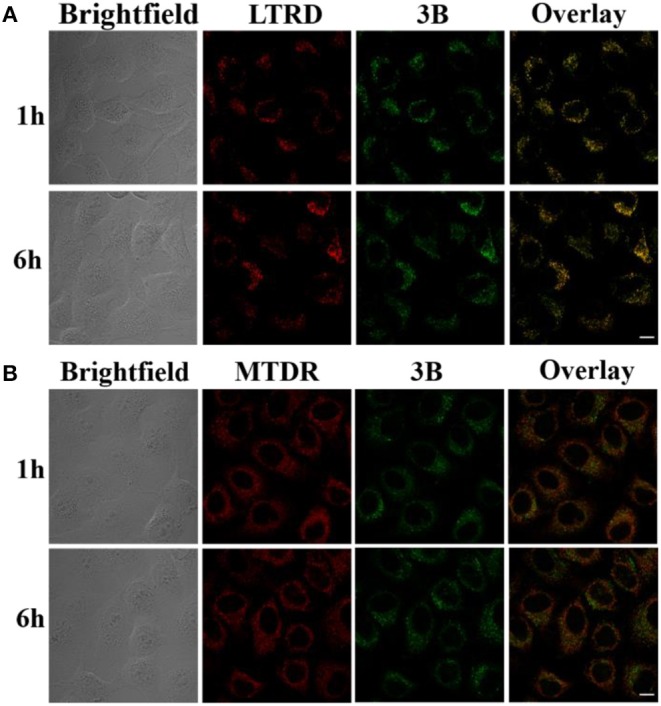
**(A)** Confocal images of A549 cells labeled by complex **3B** (10 μM) and LTRD (75 nM, 1 h) after 1 h and 6 h (collected from 549 to 651 nm and excited at 594 nm). **(B)** Confocal images of A549 cells labeled by complex **3B** (10 μM) and MTDR (500 nM, 30 min) after 1 h and 6 h (collected from 610 to 756 nm and excited at 644 nm). Complex **3B** is excited at 488 nm. Scale bar: 20 μm.

The co-localization of Ir^III^ NHC complexes was observed by laser confocal microscopy. Lyso Tracker Red DND-99 (LTRD) and Mito Tracker Deep Red (MTDR) were used. As shown in Figure 6, complex **3B** was assimilated effectively by the A549 cells, and the complex mainly targeted lysosomes. The Pearson's co-localization coefficients (PCCs) after 1 h and 6 h were 0.70 and 0.81, respectively, however, which for mitochondria were 0.08 and 0.13. As the waste disposal center, lysosomes play an important role in many physiological processes and signal transduction pathways (Boya and Kroemer, 2008). These organelles are acidic (pH 4.5–5.5) and contain abundant nitrogen atoms (containing lone electron pairs). Thus, NHC pro-ligands provide convenient conditions for the lysosomes targeting. Interestingly, the Ir^III^ NHC complex did not lead immediately induced A549 cells death. This feature is convenient for observing the effects the complexes on lysosome morphology.

**Figure 7:**
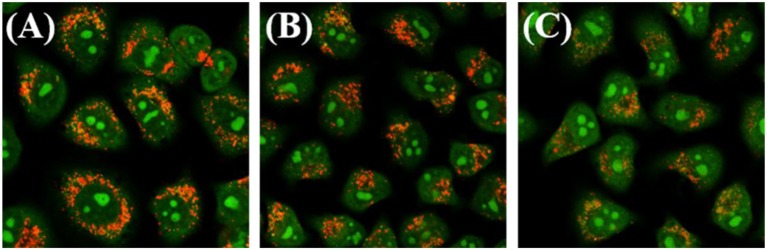
Observation of lysosomal disruption in A549 cells loaded with complex **3B** for 6 h at 37°C, then stained with acridine orange (AO) (5 μM) at 37°C for 15 min. The cells were treated with **(A)** only AO; **(B)** AO and complex **3B** (1.0 × IC_50_); **(C)** AO and complex **3B** (2.0 × IC_50_). Data were collected at 510 ± 20 nm (green) and 625 ± 20 nm (red) after excited at 488 nm. Scale bar: 20 μm.

The destruction of the lysosomal membrane destroys the integrity of the lysosome and induces cell death (Saftig and Klumperman, 2009; Zhitomirsky and Assaraf, 2017). Acridine orange (AO), an effective staining material for acidic organelles, was used to detect lysosomal damage induced by complex **3B**. Then, whether the damage is the result of lysosome targeting specificity was determined. Green fluorescence represents the binding of AO to RNA in the nucleus or cytoplasm, whereas red fluorescence shows aggregation in the lysosome (Yamabe et al., 2016). As shown in Figure 7, A549 cells subjected to AO alone showed significant red fluorescence in their lysosomes, but the intensity of the red fluorescence decreased quickly after the cells were exposed to complex **3B** with concentrations of 1.0 × IC_50_ and 2.0 × IC_50_ for 6 h, which was the result of lysosomal damage, which was mainly attributed to the introduction of nitrogen-containing ligands into the complexes. The ligands increased the total alkalinity of the molecules, which easily accumulated and damaged the integrity of the acidic lysosomes (Daum et al., 2017). Additionally, the intracellular iridium contents of **3B** in the cytosol, nucleus, nuclear chromatin and cytoskeleton fractions isolated from A549 cells were determined by inductively coupled plasma mass spectrometry (ICP-MS) after 24 h exposure. **3B** is mainly accumulated in the cytosol (Table S8), which further confirm the results of lysosomal damage. Meanwhile, all these explains why these complexes did not target the mitochondria, but the fact that mitochondrial dysfunction occurred, including the elevation of intracellular ROS levels and the depolarization of MMP. The results indicated that Ir^III^ NHC complexes can induce apoptosis by lysosomal damage.

**Figure 8:**
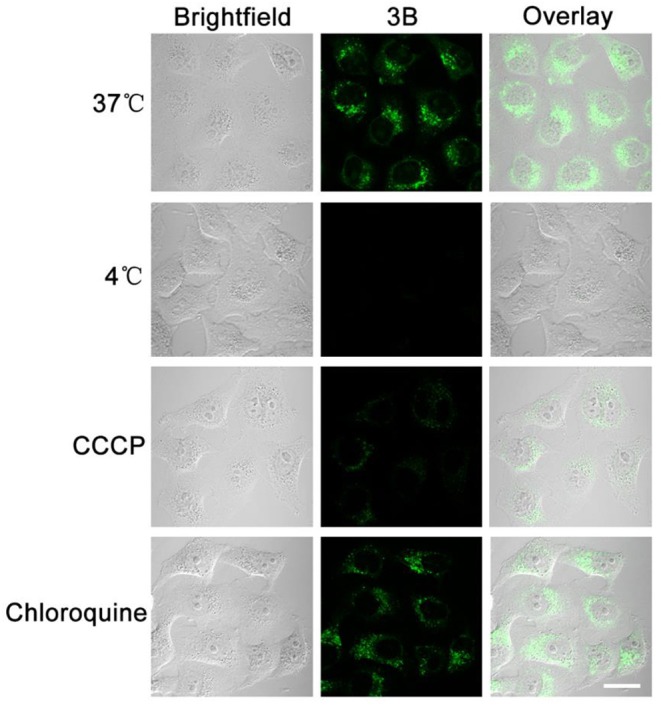
A549 cells were cultured at 37, 4°C, metabolic inhibitor (CCCP, 50 μM) and chloroquine (50 μM), and the uptake of complex **3B** (10 μM, 30 min) was observed by confocal microscopy. The fluorescence was excited at 488 nm and collected from 549 nm to 651 nm. Scale bar: 20 μm.

Small molecule drugs permeate cell membranes through different modes of action, mainly including energy-independent and energy-dependent mechanisms (Li et al., 2011). When the A549 cells were pretreated at 4°C or with carbonyl cyanide 3-chlorophenyl-hydrazone (CCCP, a metabolic inhibitor), their uptake efficiency decreased considerably after they were incubated with complex **3B** (Figure 8), although no considerable change was observed in the level for complex **3B** after pretreatment at 37°C or with chloroquine (an endocytosis inhibitor, which could inhibit endoderm acidification). Therefore, we conclude that the target Ir^III^ complex enters cells through an energy-dependent mechanism, including hydrophobic factors, which are also important in entering cells (Ye et al., 2017).

## Conclusions

A series of half-sandwich Ir^III^ NHC complexes [(η^5^-Cp^x^)Ir(C^∧^N)Cl]Cl were synthetized and characterized. Each C^∧^N chelating ligand formed a six-member ring with a metal center (Ir^III^), and the existence of benzene ring connected imidazole ring at different locations can effectively enhanced the anticancer activity of the complexes. All the complexes showed potential anticancer activity toward A549 cells, and had better anticancer activity than *cis*-platin (the clinical anticancer drug). Moreover, the complexes interacted with BSA by static quenching mode, catalyzed the conversion of NADH to NAD^+^, induced ROS production, changed mitochondrial membrane potential, disrupted the cell cycle, and eventually induced apoptosis. The laser confocal test showed that complexes entered the cells followed through an energy-dependent mechanism, effectively targeted lysosomes, further destroyed the integrity of the lysosomes, and led to cell death. These results prompted us to further explore the medicinal value of the half-sandwich Ir^III^ complex with additional NHC.

## Experimental Section

### General Procedure

The imidazole, benzimidazole, diphenylmethyl chloride, benzyl chloride, *N*-methyl-1, 2-phenylenediamine, diphenyl methane, ditertbutyl peroxide and the appropriate dimer [(η^5^-Cp^x^)IrCl_2_]_2_ includes [(η^5^-C_5_Me_5_)IrCl_2_]_2_ and [(η^5^-C_5_Me_4_-C_6_H_4_C_6_H_5_)IrCl_2_]_2_. 2-(chloromethyl)-1-methyl benzimidazol, 1-Diphenyl- methylimidazole, 1-Benzylbenzimidazole, 1-Benzhydryl-benzimidazole were prepared according to literature procedures (specific synthesis steps and experimental data in support information) (Nobre and Monteiro, 2004; Corberán et al., 2006). Nitrogen was used as the inert gas filling.

### Synthesis of Chelating Ligands

1-diphenylmethylimidazole (1.17 g, 5.0 mmol) was added to a solution of 2-chloromethyl-1-mehylbenzenimdazole (0.48 g, 2.5 mmol) in acetonitrile. The reaction solution was distilled to 10 mL by vacuum rotary evaporation after reflex for 48 h, and 10 mL ether was added to precipitate the product. Then white powder product (**L**_**1**_) was filtered, washed with ether three times and dried in air. Yield: 76.6%. ^1^H NMR (500 MHz, DMSO-*d*_6_) δ 9.37 (s, 1H, NC*H*N), 7.97 (s, 1H, imidazole-*H*), 7.84 (s, 1H, imidazole-*H*), 7.61 (dd, *J* = 7.6, 5.4 Hz, 2H, Ar-*H*), 7.47 (dq, *J* = 14.4, 7.1 Hz, 6H, Ar-*H*), 7.30 (dd, *J* = 14.5, 7.5 Hz, 6H, Ar-*H*), 7.23 (*t, J* = 7.6 Hz, 1H, NC*H*), 5.89 (s, 2H, NC*H*_2_C), 3.85 (s, 3H, NC*H*_3_).

Chelating ligands **L**_**2**_ and **L**_**3**_ were synthesized using 1-benzylbenzimidazole and 1-benzhydryl-benzimidazole with 2-chloromethyl-1-mehylbenzenimdazole by the same method, respectively, the data were as follows:

**L**_**2**_: Yield: 72.1%. ^1^H NMR (500 MHz, DMSO-*d*_6_) δ 10.22 (s, 1H, NC*H*N), 8.14–8.10 (m, 1H, Ar-*H*), 8.05–8.00 (m, 1H, Ar-*H*), 7.69–7.66 (m, 2H, Ar-*H*), 7.64 (d, *J* = 8.1 Hz, 1H, Ar-*H*), 7.56 (d, *J* = 7.1 Hz, 2H, Ar-*H*), 7.53 (d, *J* = 8.0 Hz, 1H, Ar-*H*), 7.45 (*t, J* = 7.3 Hz, 2H, Ar-*H*), 7.40 (d, *J* = 7.2 Hz, 1H, Ar-*H*), 7.32–7.28 (m, 1H, Ar-*H*), 7.22–7.18 (m, 1H, Ar-*H*), 6.28 (s, 2H, NC*H*_2_), 5.91 (s, 2H, NC*H*_2_C), 3.97 (s, 3H, NC*H*_3_).

**L**_**3**_: Yield: 72.3%. ^1^H NMR (500 MHz, DMSO-*d*_6_) δ 9.69 (s, 1H, NC*H*N), 8.13 (d, *J* = 8.1 Hz, 1H, NC*H*), 7.78 (d, *J* = 8.1 Hz, 1H, Ar-*H*), 7.73 (s, 1H, Ar-*H*), 7.70–7.61 (m, 3H, Ar-*H*), 7.52 (*t, J* = 7.9 Hz, 5H, Ar-*H*), 7.49–7.42 (m, 6H, Ar-*H*), 7.28 (*t, J* = 7.7 Hz, 1H, Ar-*H*), 7.20 (*t, J* = 7.6 Hz, 1H, Ar-*H*), 6.23 (s, 2H, NC*H*_2_C), 3.91 (s, 3H, NC*H*_3_).

### Synthesis of the Complexes (1A−3B)

General method: In a round bottom flask, silver oxide (2.4 eq) and (**L**_**1**_**-L**_**3**_) (2.0 eq) were added to a solvent of dichloromethane. After 8 h, the mixture was filtered through celite. The combined filtrates were added dropwise to a solution of dichloromethane containing Dimer ([(η^5^-Cp^x^)IrCl_2_]_2_, 1.0 eq) (Weaver et al., 2011). The solution was stirred for 8 h at room temperature, filtered with celite and washed with dichloromethane for three times. The solvent was removed by rotary evaporator and yellowish solids were obtained by crystallization in the solution of 10% dichloromethane/50% *n*-hexane. The data were listed as follows:

[(η^5^-Cp^*^)Ir(**L**_**1**_)Cl]Cl (**1A**): Yield: 65.4%. ^1^H NMR (500 MHz, DMSO-*d*_6_) δ 7.83–7.78 (m, 2H, imidazole-*H*), 7.73 (d, *J* = 7.9 Hz, 1H, NC*H*), 7.52–7.40 (m, 8H, Ar-*H*), 7.32 (d, *J* = 2.1 Hz, 1H, Ar-*H*), 7.24 (dd, *J* = 5.1, 1.7 Hz, 3H, Ar-*H*), 7.20 (d, *J* = 7.4 Hz, 2H, Ar-*H*), 6.00 (d, *J* = 16.6 Hz, 1H, NC*H*_2_C), 5.06 (d, *J* = 16.6 Hz, 1H, NC*H*_2_C), 4.08 (s, 3H, NC*H*_3_), 1.44 (s, 15H, Cp^*^-*H*). ^13^C NMR (126 MHz, DMSO) δ 154.03 (*C*-Ir), 149.42 (N*C*N), 140.79, 139.79, 138.37, 135.27, 130.09, 129.13, 128.72, 128.53, 127.75, 124.76, 124.37, 123.73, 122.47, 119.18, 112.71, 90.97, 64.96, 45.04, 31.87, 9.45 ppm. Elemental analysis: Found: C, 54.13; H, 4.79; N, 7.24%, calcd for C_35_H_37_Cl_2_IrN_4_: C, 54.12; H, 4.80; N, 7.21%. ESI-MS (*m/z*): calcd for C_35_H_37_ClIrN_4_: 741.23 [M-Cl]^+^; Found: 741.01.

[(η^5^-Cp^*^)Ir(**L**_**2**_)Cl]Cl (**2A**): Yield: 59.2%. ^1^H NMR (500 MHz, DMSO-*d*_6_) δ 8.23 (d, *J* = 8.3 Hz, 1H, Ar-*H*), 7.83 (d, *J* = 8.0 Hz, 1H, Ar-*H*), 7.66 (d, *J* = 7.9 Hz, 1H, Ar-*H*), 7.47 (dd, *J* = 11.2, 4.1 Hz, 1H, Ar-*H*), 7.44–7.38 (m, 2H, Ar-*H*), 7.26 (s, 5H, Ar-*H*), 7.20 (*t, J* = 7.4 Hz, 1H, Ar-*H*), 6.98 (d, *J* = 8.2 Hz, 1H, Ar-*H*), 6.28 (d, *J* = 17.0 Hz, 1H, NC*H*_2_), 6.00 (d, *J* = 15.3 Hz, 1H, NC*H*_2_), 5.78 (d, *J* = 15.4 Hz, 1H, NC*H*_2_C), 5.29 (d, *J* = 16.9 Hz, 1H, NC*H*_2_C), 4.20 (s, 3H, NC*H*_3_), 1.70 (s, 15H, Cp^*^-*H*). ^13^C NMR (126 MHz, DMSO) δ 148.66 (*C*-Ir), 138.56 (N*C*N), 136.40, 135.28, 135.09, 128.67, 128.03, 123.90, 112.92, 112.75, 92.56, 91.70, 52.30, 32.06, 9.39, 8.72 ppm. Elemental analysis: Found: C, 52.74; H, 4.71; N, 7.49%, calcd for C_33_H_35_Cl_2_IrN_4_: C, 52.79; H, 4.70; N, 7.46%. ESI-MS (*m/z*): calcd for C_33_H_35_ClIrN_4_: 715.22 [M-Cl]^+^; Found: 714.95.

[(η^5^-Cp^*^)Ir(**L**_**3**_)Cl]Cl (**3A**): Yield: 67.9%. ^1^H NMR (500 MHz, DMSO-*d*_6_) δ 8.30 (d, *J* = 8.4 Hz, 1H, Ar-*H*), 7.98 (s, 1H, NC*H*), 7.83 (d, *J* = 8.1 Hz, 1H, Ar-*H*), 7.77 (d, *J* = 8.0 Hz, 1H, Ar-*H*), 7.50 (dd, *J* = 22.0, 7.0 Hz, 4H, Ar-*H*), 7.45–7.41 (m, 1H, Ar-*H*), 7.37 (*t, J* = 7.7 Hz, 1H, Ar-*H*), 7.31 (d, *J* = 7.8 Hz, 2H, Ar-*H*), 7.24–7.14 (m, 5H, Ar-*H*), 7.07 (*t, J* = 8.0 Hz, 1H, Ar-*H*), 6.72 (d, *J* = 8.3 Hz, 1H, Ar-*H*), 6.29 (d, *J* = 16.8 Hz, 1H, NC*H*_2_C), 5.21 (d, *J* = 16.9 Hz, 1H, NC*H*_2_C), 4.21 (s, 3H, NC*H*_3_), 1.46 (s, 15H, Cp^*^-*H*). ^13^C NMR (126 MHz, DMSO) δ 156.63 (*C*-Ir), 150.24 (N*C*N), 146.24, 141.10, 134.17, 133.79, 133.12, 132.15, 127.18, 126.55, 123.28, 122.14, 118.90, 66.23, 48.65, 34.79, 13.46 ppm. Elemental analysis: Found: C, 56.61; H, 4.78; N, 6.79 %, calcd for C_39_H_39_Cl_2_IrN_4_: C, 56.65; H, 4.75; N, 6.78 %. ESI-MS (*m/z*): calcd for C_39_H_39_ClIrN_4_: 789.55 [M-Cl]^+^; Found: 789.01.

[(η^5^-Cp^xbiph^)Ir(**L**_**1**_)Cl]Cl (**1B**): Yield: 66.1%. ^1^H NMR (500 MHz, DMSO-*d*_6_) δ 7.85–7.71 (m, 9H, Ar-*H*), 7.52 (*t, J* = 7.7 Hz, 2H, imidazole-*H*), 7.49–7.45 (m, 1H, NC*H*), 7.41 (dd, *J* = 14.4, 7.1 Hz, 2H, Ar-*H*), 7.35 (d, *J* = 2.1 Hz, 1H, Ar-*H*), 7.32 (dd, *J* = 6.9, 2.8 Hz, 2H, Ar-*H*), 7.24–7.17 (m, 4H, Ar-*H*), 7.11 (s, 1H, Ar-*H*), 7.05 (*t, J* = 7.8 Hz, 2H, Ar-*H*), 6.48 (d, *J* = 7.7 Hz, 2H, Ar-*H*), 6.03 (d, *J* = 16.7 Hz, 1H, NC*H*_2_C), 5.14 (d, *J* = 16.6 Hz, 1H, NC*H*_2_C), 4.10 (s, 3H, NC*H*_3_), 1.90 (s, 3H, Cp^xbiph^-*H*), 1.64 (s, 3H, Cp^xbiph^-*H*), 1.43 (s, 3H, Cp^xbiph^-*H*), 1.34 (s, 3H, Cp^xbiph^-*H*). ^13^C NMR (126 MHz, DMSO) δ 152.57 (*C*-Ir), 149.12 (N*C*N), 140.73, 140.41, 139.89, 139.34, 138.05, 135.34, 131.39, 130.75, 129.83, 129.62, 128.59, 128.29, 127.81, 127.42, 127.06, 124.85, 124.37, 123.73, 122.53, 119.47, 112.79, 101.14, 97.67, 97.03, 83.35, 80.92, 65.25, 31.88, 11.37, 9.95, 9.69 ppm. Elemental analysis: Found: C, 60.35; H, 4.72; N, 6.10%, calcd for C_46_H_43_Cl_2_IrN_4_: C, 60.38; H, 4.74; N, 6.12%. ESI-MS (*m/z*): calcd for C_46_H_43_ClIrN_4_: 879.28 [M-Cl]^+^; Found: 879.43.

[(η^5^-Cp^xbiph^)Ir(**L**_**2**_)Cl]Cl (**2B**): Yield: 63.7%. ^1^H NMR (500 MHz, DMSO-*d*_6_) δ 8.25 (d, *J* = 8.4 Hz, 1H, Ar-*H*), 7.84 (d, *J* = 8.3 Hz, 1H, Ar-*H*), 7.76–7.64 (m, 7H, Ar-*H*), 7.59 (d, *J* = 8.2 Hz, 1H, Ar-*H*), 7.56–7.36 (m, 6H, Ar-*H*), 7.32 (*t, J* = 7.6 Hz, 1H, Ar-*H*), 7.22–7.10 (m, 3H, Ar-*H*), 6.97–6.90 (m, 2H, Ar-*H*), 6.32 (d, *J* = 17.0 Hz, 1H, NC*H*_2_), 5.80 (d, *J* = 15.7 Hz, 1H, NC*H*_2_), 5.42 (d, *J* = 15.8 Hz, 1H, NC*H*_2_C), 5.35 (d, *J* = 17.0 Hz, 1H, NC*H*_2_C), 4.22 (s, 3H, NC*H*_3_), 1.90 (s, 2H, Cp^xbiph^-*H*), 1.83 (d, *J* = 11.3 Hz, 3H, Cp^xbiph^-*H*), 1.74 (s, 3H, Cp^xbiph^-*H*), 1.70 (d, *J* = 9.8 Hz, 4H, Cp^xbiph^-*H*). ^13^C NMR (126 MHz, DMSO) δ 167.58 (*C*-Ir), 148.68 (N*C*N), 138.49, 136.40, 135.28, 135.09, 133.85, 128.67, 128.03, 124.81, 123.83, 119.19, 112.84, 112.59, 92.56, 91.70, 52.30, 42.04, 32.08, 31.43, 22.54, 14.45, 9.39 ppm. Elemental analysis: Found: C, 59.41; H, 4.66; N, 6.29%, calcd for C_44_H_41_Cl_2_IrN_4_: C, 59.45; H, 4.65; N, 6.30 %. ESI-MS (*m/z*): calcd for C_44_H_41_ClIrN_4_: 853.26 [M-Cl]^+^; Found: 853.32.

[(η^5^-Cp^xbiph^)Ir(**L**_**3**_)Cl]Cl (**3B**): Yield: 69.0%. ^1^H NMR (500 MHz, DMSO-*d*_6_) δ 8.31 (d, *J* = 8.5 Hz, 1H, Ar-*H*), 7.86 (d, *J* = 8.2 Hz, 1H, NC*H*), 7.81 (d, *J* = 7.2 Hz, 2H, Ar-*H*), 7.73 (dd, *J* = 13.9, 6.8 Hz, 6H, Ar-*H*), 7.54 (*t, J* = 7.7 Hz, 2H, Ar-*H*), 7.43 (ddt, *J* = 28.1, 12.8, 7.4 Hz, 5H, Ar-*H*), 7.27 (*t, J* = 7.4 Hz, 1H, Ar-*H*), 7.18 (dd, *J* = 8.8, 4.6 Hz, 1H, Ar-*H*), 7.14 (d, *J* = 4.3 Hz, 3H, Ar-*H*), 7.05 (dd, *J* = 15.6, 7.7 Hz, 3H, Ar-*H*), 6.58 (dd, *J* = 8.0, 4.2 Hz, 3H, Ar-*H*), 6.32 (d, *J* = 16.9 Hz, 1H, NC*H*_2_C), 5.28 (d, *J* = 16.8 Hz, 1H, NC*H*_2_C), 4.24 (s, 3H, NC*H*_3_), 1.89 (s, 3H, Cp^xbiph^-*H*), 1.69 (s, 3H, Cp^xbiph^-*H*), 1.39 (s, 3H, Cp^xbiph^-*H*), 1.33 (s, 3H, Cp^xbiph^-*H*). ^13^C NMR (126 MHz, DMSO) δ 165.72 (*C*-Ir), 149.11 (N*C*N), 140.46, 139.88, 138.92, 138.21, 136.57, 135.55, 135.16, 134.01, 131.11, 130.74, 129.66, 129.26, 128.90–128.18, 127.99, 127.65, 127.45, 127.15, 124.86, 123.87, 119.26, 114.68, 113.22, 112.79, 102.22, 98.12, 97.38, 85.56, 65.56, 63.28, 42.16, 32.28, 31.43, 22.54, 11.11, 9.81 ppm. Elemental analysis: Found: C, 62.24; H, 4.69; N, 5.84 %, calcd for C_50_H_45_Cl_2_IrN_4_: C, 62.23; H, 4.70; N, 5.81%. ESI-MS (*m/z*): calcd for C_50_H_45_ClIrN_4_: 929.24 [M-Cl]^+^; Found: 929.40.

## Data Availability Statement

All datasets generated for this study are included in the article/Supplementary Material.

## Author Contributions

XL, YH, and XG conceived the research and conducted the experiments. ZL directed the project and co-wrote the paper.

### Conflict of Interest

The authors declare that the research was conducted in the absence of any commercial or financial relationships that could be construed as a potential conflict of interest.
