# Supplementary material for: Imidazole and Benzimidazole Modified Half-Sandwich IridiumIII N-Heterocyclic Carbene Complexes: Synthesis, Anticancer Application, and Organelle Targeting
Source: Front Chem. 2020 Mar 17;8:182. doi: 10.3389/fchem.2020.00182 (PMC7090125; doi:10.3389/fchem.2020.00182)
Supplement: Supplementary file 1 [file Data_Sheet_1.docx]

Supplementary Material

# Supplementary Data

**Materials and** **Instrumentation**

All synthesis operations were carried out in a nitrogen atmosphere. IrCl_3_·nH_2_O available from Sigma -Aldrich. For biological experiments, BSA, DMEM medium, fetal bovine serum, penicillin/ streptomycin mixture, trypsin/EDTA, cisplatin, MTT and phosphate buffered saline (PBS) were purchased from Sangon Biotech. Test compounds are dissolved in DMSO and diluted in tissue culture medium prior to use. Stock solutions of cisplatin (10 mM) and complexes **1A**-**3B** (10 mM) were prepared in DMSO. All stock solutions were stored at -20 °C, thawed and diluted with medium before each experiment.

**NMR Spectroscopy** ^1^H NMR spectra were acquired in 5 mm NMR tubes at 298 K on Bruker DPX 500 (^1^H = 500.13 MHz) spectrometers, using CDCl_3_ (7.26 ppm), DMSO-*d*_6_ (2.50 ppm) as solvents. All data processing was carried out using XWINNMR version 3.6 (Bruker UK Ltd.).

**UV-Vis Spectroscopy** UV-Vis spectrum of the compound recorded by TU-1901 UV spectrophotometer is the 1 cm path (volume 3 mL) of quartz cuvette. Spectral software is processed using UV Winlab. Unless otherwise specified, experiments were performed at 298 K.

**Cytotoxicity Test** After plating 5000 A549 cells per well in 96-well plates, the cells were preincubated in drug-free media at 310 K for 24 h before adding different concentrations of the compounds to be tested. In order to prepare the stock solution of the drug, the solid complex was dissolved in DMSO. This stock was further diluted using cell culture medium until working concentrations were achieved. The drug exposure period was 24 h. Subsequently, 15 µL of 5 mg mL^-1^ MTT solution was added to form a purple formazan. Afterwards, 100 µL of dimethyl sulfoxide (DMSO) was transferred into each well to dissolve the purple formazan, and results were measured using a microplate reader (DNM-9606, Perlong Medical, Beijing, China) at an absorbance of 570 nm. Each well was triplicated and each experiment repeated at least three times. IC_50_ values quoted are mean ± SEM.

**LogP Determination** Octanol-saturated water (OSW) and water-saturated octanol (WSO) were prepared using analytical grade octanol and 0.2 M aqueous NaCl solution (to suppress hydrolysis of the chloride complexes). Aliquots of stock solutions of iridium complexes in OSW were added to equal volumes of WSO and shaken in an IKA Vibrax VXC basic shaker for 4 h at 500 g/min to allow partition at ambient temperature (~298 K). The aqueous layer was carefully separated from the octanol layer for iridium analysis. Ir was quantified from aliquots taken from the octanol-saturated aqueous samples before and after partition. Partition coefficients of Ir^III^ complexes were calculated using the equation logP = log (Ir_WSO_/Ir_OSW_), where Ir_WSO_ was obtained by subtraction of the Ir content of the aqueous layer after partition from the Ir content of the aqueous layer before partition.

**Hydrolysis studies** Solutions of complex **3B** with final concentrations of 1 mM in 50% CD_3_OD-*d*_4_/50% D_2_O (*v/v*) was prepared by dissolution of the complexes in CD_3_OD-*d*_4_ followed by rapid dilution with D_2_O. ^1^H NMR spectra were recorded after various time intervals at 310 K. Solutions of complex **3B** with final concentrations of 50 µM in 50% MeOH/50% H_2_O (*v/v*) was prepared by dissolution of the complexes in MeOH followed by rapid dilution with H_2_O. UV-Vis spectra of these solutions were recorded at 298 K after various time intervals.

**Reaction with NADH** The reaction of complexes **1B**-**3B** (ca. 1 µM) with NADH (ca. 100 µM) in 60% MeOH/40% H_2_O (*v/v*) was monitored by UV-Vis at 298 K after various time intervals. TON was calculated by measuring the concentration difference of NADH after 7 h divided by the concentration of iridium catalyst. The concentration of NADH was obtained using an extinction coefficient **ε** (339 nm) = 6220 M^-1^ cm^-1^.

**Binding with BSA** The titration experiments including UV-Vis absorption and fluorescence quenching were performed at a constant concentration of BSA. BSA stock solutions were prepared in Tris buffer (5 mM Tris-HCl/10 mM NaCl, pH=7.2) and stored at 4 °C. All spectra were recorded after each successive addition of compound and incubated for 5 min at room temperature to complete the interaction. The iridium complex was added to the sample cuvette and the reference cuvette to counteract its absorption of the iridium complex in the ultraviolet region (the "internal filter effect"). Fluorescence emission spectra of BSA excited at 285 nm were recorded in the absence and presence of an iridium complex. Synchronous fluorescence spectra of BSA with different concentrations of the complexes were obtained from 240 to 500 nm when *Δλ* = 60 nm and *Δλ* = 15 nm.

With the increase of iridium complex concentration, BSA solution was titrated and the fluorescence intensity of BSA solution at 343 nm decreased gradually, which indicated that two complexes could interact with BSA through static quenching mode. The possible quenching mechanism can be explained by the classical Stern-Volmert equation (1):

*F_0_/F* = 1 + *K_sv_* [Q] = 1 + *K_q_τ_0_* [Q] (1)

where *F_0_* and *F* are the fluorescence intensities in the absence and in the presence of quencher Q, [Q] is the concentration of the quencher, *K_q_* is the quenching rate constant and *τ_0_* is the average lifetime of the fluorescent material in the absence of quencher The value of about 10^-8^ s. *K_sv_* is Stern-Volmert constant, which can be obtained from the ratio of the slope to the intercept of the *F_0_****/****F* curve to the concentration of the tested complexes (**Supporting Figure 9 (A-C)**). The value of *K_q_* indicates that the static quenching mechanism dominates in the interaction between the iridium complex and BSA. The binding constant *K_b_* and the number of complexes bound to BSA (n) were calculated using the equation (2) (**Supporting Figure 9** (D-F)):

log [(*F_0_*-*F*)*/F*] = log *K_b_* + n log [Q] (2)

The *K_b_* and *K_q_* sizes of the two complexes were 10^4^ M^-1^ and 10^12^ M^-1^ s^-1^, respectively, indicating that the complex has the ability to bind BSA.

**Cell cycle analysis** The A549 cancer cells at 1.5 × 10^6^ per well were seeded in a six-well plate. Cells were preincubated in drug-free media at 310 K for 24 h, after which complex **3B** were added at concentrations of 0.25 × IC_50_, 0.5 × IC_50_, 1.0 × IC_50_ and 2.0 × IC_50_ of complex **3B** against A549 cancer cells. After 24 h of drug exposure, supernatants were removed by suction and cells were washed with PBS. Finally, cells were harvested using trypsin-EDTA and fixed for 24 h using cold 70 % ethanol. DNA staining was achieved by resuspending the cell pellets in PBS containing propidium iodide (PI) and RNAse. Cell pellets were washed and resuspended in PBS before being analyzed in a flow cytometer (ACEA NovoCyte, Hangzhou, China) using excitation of DNA-bound PI at 488 nm, with emission at 585 nm. Data were processed using Novo Express™ software. The cell cycle distribution is shown as the percentage of cells containing G_0_/G_1_, S and G_2_/M DNA as identified by propidium iodide staining.

**ROS determination** Flow cytometry analysis of ROS generation in the cells caused by exposure to iridium complexes was carried out using the Reactive Oxygen Species Assay Kit (Beyotime Institute of Biotechnology, Shanghai, China) according to the supplier instructions. Briefly, 1.5×10^6^ A549 cancer cells per well were seeded in a six-well plate. Cells were preincubated in drug-free media at 310 K for 24 h in a 5% CO_2_ humidified atmosphere, and then complex **3B** was added at concentrations of 0.25×IC_50_ and 0.5×IC_50_ of complex **3B** against A549 cancer cells. After 24 h of drug exposure, cells were washed twice with PBS and then incubated with the DCFH-DA probe (10 µM) at 37 °C for 30 min, and then washed triple immediately with PBS. The fluorescence intensity was analyzed by flow cytometry (ACEA NovoCyte, Hangzhou, China). Data were processed using NovoExpress™ software. At all times, samples were kept under dark conditions to avoid light-induced ROS production.

**Mitochondrial membrane potential** Analysis of the changes of mitochondrial potential in cells after exposure to iridium complexes was carried out using the Mitochondrial membrane potential assay kit with JC-1 (Beyotime Institute of Biotechnology, Shanghai, China) according to the manufacturer’s instructions. Briefly, 1.5 × 10^6^ A549 cancer cells were seeded in six-well plates left to incubate for 24 h in drug-free medium at 310 K in a humidified atmosphere. Drug solutions, at concentrations of 0.25×IC_50_, 0.5×IC_50_, 1.0×IC_50_ and 2.0×IC_50_ of complex **3B** against A549 cancer cells, were added in triplicate, and the cells were left to incubate for a further 24 h under similar conditions. Supernatants were removed by suction, and each well was washed with PBS before detaching the cells using trypsin-EDTA. Staining of the samples was done in flow cytometry tubes protected from light, incubating for 30 min at ambient temperature. The samples were immediately analyzed by a flow cytometer (ACEA NovoCyte, Hangzhou, China). For positive controls, the cells were exposed to carbonyl cyanide 3-chlorophenyl-hydrazone, CCCP (5 μM), for 20 min. Data were processed using NovoExpress™ software.

**Induction of apoptosis** In order to investigate whether the reduction in cell viability observed in the MTT assay is based on apoptosis, A549 cells were treated with complex **3B** at 0.5, 1.0 and 2.0 equipotent concentrations of IC_50_ for 24 h, and then stained with Annexin V/PI and analyzed by flow cytometry. This allowed determination of cell populations as viable (unstained, only self-fluorescence), early apoptosis (stained by Annexin Vonly, green fluorescence), late apoptosis (stained by Annexin V and PI, green and red fluorescence), and nonviable (stained by PI only, red fluorescence). To put it simply, A549 cells (1.5×10^6^) were seeded into a six-well plate. The cells were preincubated in drug-free medium at 310 K for 24 h, after which drugs were added at concentrations of 0.5×IC_50_, 1.0×IC_50_ and 2.0×IC_50_. After 24 h of drug exposure, the cells were collected, washed once with PBS, and resuspended in 195 μL of Annexin V-FITC binding buffer, which was then added to 5 μL of Annexin V-FITC and 10 μL of PI, and then incubated at room temperature in the dark for 15 min. Subsequently, the buffer was placed in an ice bath in the dark. The samples were analyzed by a flow cytometer (ACEA NovoCyte, Hangzhou, China).

**Cellular uptake and cell imaging** A549 cells were incubated with Ir^III^ complex **3B** (10 μM) and Lyso Tracker Red DND-99 (LTRD, 75 nM) at 37 °C for 30 min. The cells were washed three times with PBS and immediately observed with a confocal microscope. The excitation wavelength of the Ir^III^ complex is 488 nm and the collection wavelength is 549-651 nm. The excitation wavelength of Lyso Tracker Red DND-99 (LTRD) is 594 nm and the collection wavelength is 493-630 nm.

Small molecules can penetrate cell membrane through different mechanisms including energy-dependent (e.g. endocytosis and active transport) and energy-independent (e.g. facilitated diffusion and passive diffusion) pathways. Endocytosis is generally the general mechanism of extracellular substances entering the cell and is an energy-dependent uptake pathway that can be hindered by hypothermia or adenosine triphosphate (ATP) depletion. 3-Chlorophenylhydrazone (CCCP) and chloroquine were used as energy inhibitors and endocytosis inhibitors, respectively. A549 cells were seeded in 35 mm dishes for 24 h and preincubated with CCCP (50 μM) or chloroquine (50 μM) for 1 h. The medium was removed and the cells were then incubated with complex **3B** (10 μM) for 30 min. The cells were washed three times with PBS and visualize by a confocal microscope immediately.

**Lysosome damage** As a control group, no drug was added to the cells. Complex **3B** was excited at 488 nm and collected at a wavelength of 493-630 nm. Red channel is LTDR specific. A549 cells were incubated with complex **3B (**1.0 × IC_50_ and 2.0×IC_50_**)** for 6 hours, then washed twice with PBS, added Acridine orange (AO, 5 μM) for 15 minutes, then rinsed twice with PBS and laser confocal assay. Acridine Orange (AO) binds to RNA and emits in the green spectral region. Conversely, Acridine orange (AO) exhibits orange fluorescence when it accumulates in lysosomes.

**Inductively Coupled Plasma Mass Spectrometry (ICP-MS).** A549 cells were seeded in 90 mm dishes for 24 h (three dishes were prepared per compound tested). The media was removed and replaced with fresh media containing the tested complex (5 μM) for 24 h. After the removal of the culture media and rinse with 3 mL of PBS buffer (1X), the cells were treated with trypsin (2 mL, 0.25%) and centrifuged at 1000 rpm. The cells were counted, one half of the cells were centrifuged, quickly washed with PBS, and stored at 253 K for determination of total cell accumulation of iridium. Another half of the samples was used for cytosol, nucleus, nuclear chromatin and cytoskeleton, using a Subcellular Protein Fractionation Kit for Cultured Cells extraction kit (Thermo Scientific). The samples were nitrolysis with concentrated HNO_3_ at 95 °C for 2 h, H_2_O_2_ at 95 °C for 1.5 h and concentrated HCl at 37 °C for 0.5 h. Finally, the solution was diluted to 2 mL with MQ water and the iridium content was measured by the inductively coupled plasma mass spectrometer (ICP-MS; VG Elemental).

**Synthesis of 2-(chloromethyl)-1-methylbenzimidazole**

Monochloroacetic acid (1.00 g, 0.01 mol) and *N*-methyl-1, 2-phenylenediamine (6.80 g, 0.05 mol) were refluxed in 4 M HCl for 7.5 h. The reaction mixture was neutralized by K_2_CO_3_ saturated solution. The precipitated product was collected by vacuum filtration, and white solid was obtained after drying. Yield: 80.2%. ^1^H NMR (500 MHz, CDCl_3_) δ 7.77 (d, *J* = 7.9 Hz, 1H, Ar-*H*), 7.39 - 7.29 (m, 3H, Ar-*H*), 4.87 (s, 2H, NC*H*_2_C), 3.89 (s, 3H, N-C*H*_3_).

**Synthesis of 1-Diphenylmethylimidazole**

Imidazole (1.00 g, 15 mmol), KOH (1.24 g, 22 mmol), and 10 mL of DMSO were added to a round-bottomed flask. The mixture was stirred at 100 °C for 1 h, and then diphenylmethyl chloride (2.60 mL, 15 mmol) was added. After stirring overnight at 100 °C the reaction mixture was extracted with Et_2_O/H_2_O and the organic extracts were collected and dried over Na_2_SO_4_. The solvent was removed under vacuum gave oil and the product was obtained. Yield: 81.7%. ^1^H NMR (500 MHz, CDCl_3_) δ 7.39 (s, 1H, NC*H*N), 7.38 - 7.32 (m, 10H, Ar-*H*), 7.07 (s, 1H, imidazole-*H*), 6.84 (s, 1H, imidazole-*H*), 6.52 (s, 1H, NC*H*)

**Synthesis of** **1-Benzylbenzimidazole**

A solution of benzimidazole (1.18 g, 0.01 mol), benzyl chloride (1.26 g, 0.01 mol) and K_2_CO_3_ (6.89 g, 0.05 mol) in 15 mL of DMF was added to a round bottom flask. The mixture was stirred at 120 °C for 5 h, and then cooled to room temperature. After the reaction mixture were added H_2_O, and extracted with Et_2_O, and the organic extracts were collected and dried over Na_2_SO_4_. The solvent was removed under vacuum and white solid product was obtained. Yield: 76.5%.^1^H NMR (500 MHz, CDCl_3_) δ 7.95 (s, 1H, NC*H*N), 7.82 (d, *J* = 7.1 Hz, 1H, Ar-*H*), 7.34 - 7.31 (m, 2H, Ar-*H*), 7.29 (dd, *J* = 8.4, 5.3 Hz, 2H, Ar-*H*), 7.27 - 7.23 (m, 2H, Ar-*H*), 7.18 (d, *J* = 6.5 Hz, 2H, Ar-*H*), 5.36 (s, 2H, NC*H*_2_).

**Synthesis of** **1-Benzhydryl-benzimidazole**

In N_2_ atmosphere, an oven-dried Schlenk bottle equipped with magneton was charged with benzimidazole (59.00 mg, 0.5 mmol), FeCl_2_ (6.4 mg, 0.05 mmol), and diphenyl methane (504.00 mg, 3 mmol). Then, a solution of chlorobenzene in which ditertbutyl peroxide (146.0 mg, 1.0 mmol) was dissolved was added. The resulting mixture was stirred at 120 °C for 24 h. After cooling to room temperature, the solvent was removed under reduced pressure. The residue was purified by column chromatography eluting with ethyl acetate/petroleum (1:2), and white solid product was obtained. Yield: 72.4%. ^1^H NMR (500 MHz, CDCl_3_) δ 7.85 (d, *J* = 8.1 Hz, 1H, NC*H*N), 7.66 (s, 1H, Ar-*H*), 7.38 (dd, *J* = 5.2, 1.8 Hz, 6H, Ar-*H*), 7.30 (t, *J* = 7.7 Hz, 1H, Ar-*H*), 7.22 (t, *J* = 7.6 Hz, 1H, Ar-*H*), 7.19 - 7.12 (m, 5H, Ar-*H*), 6.77 (s, 1H, NC*H*).

# Supplementary Figures


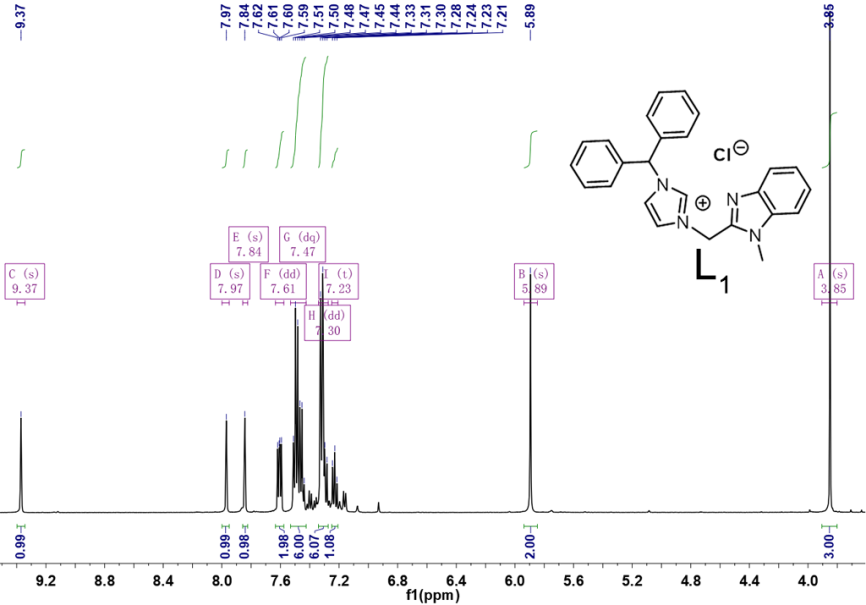


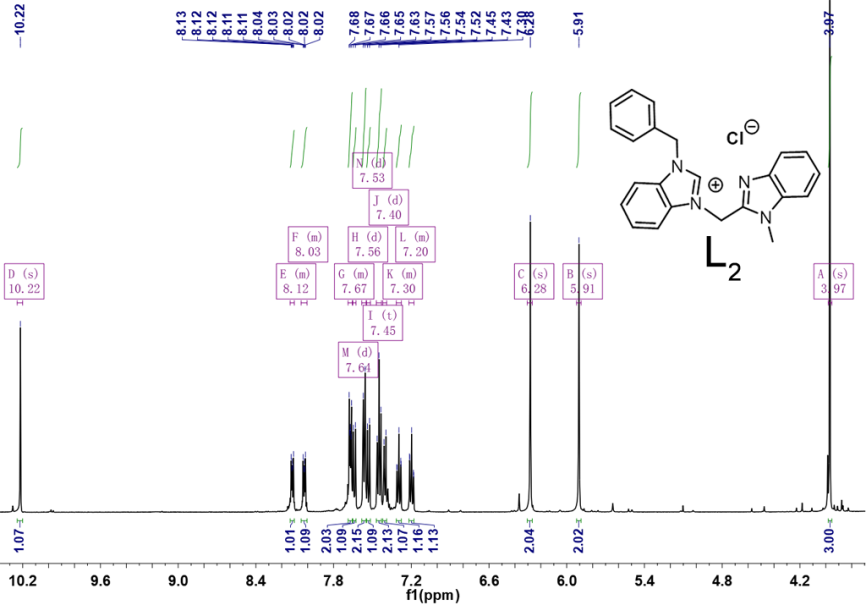


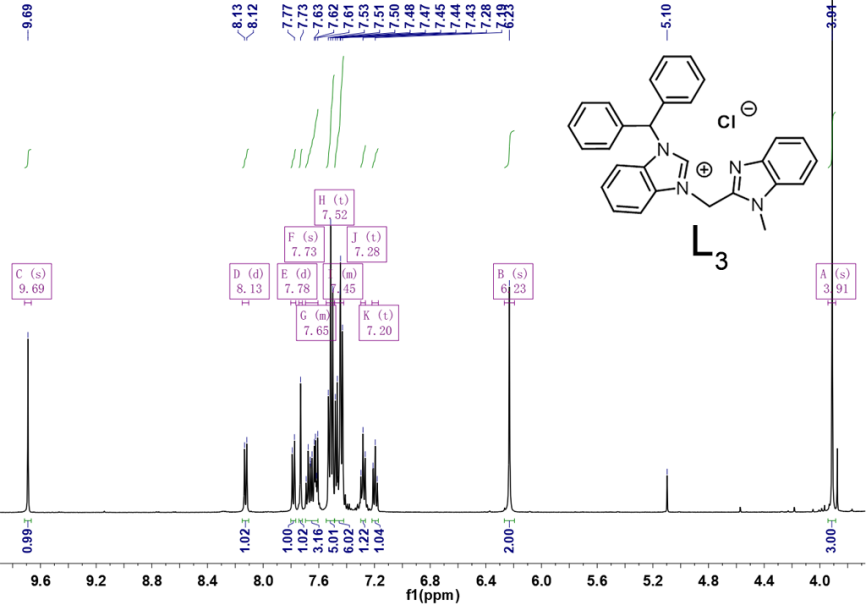


**Supplementary Figure 1** The ^1^H NMR (500.13 MHz, DMSO) peak integrals of **L_1_-L_3_**.


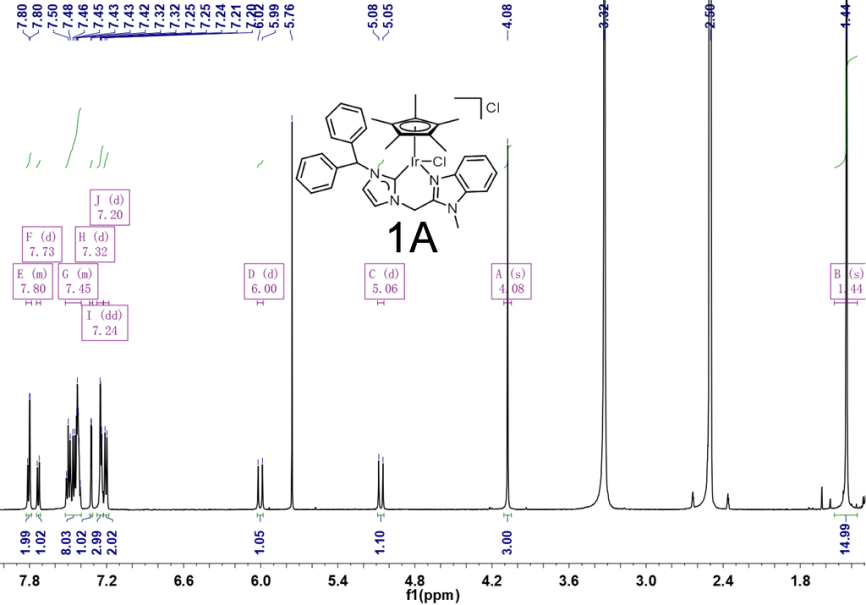


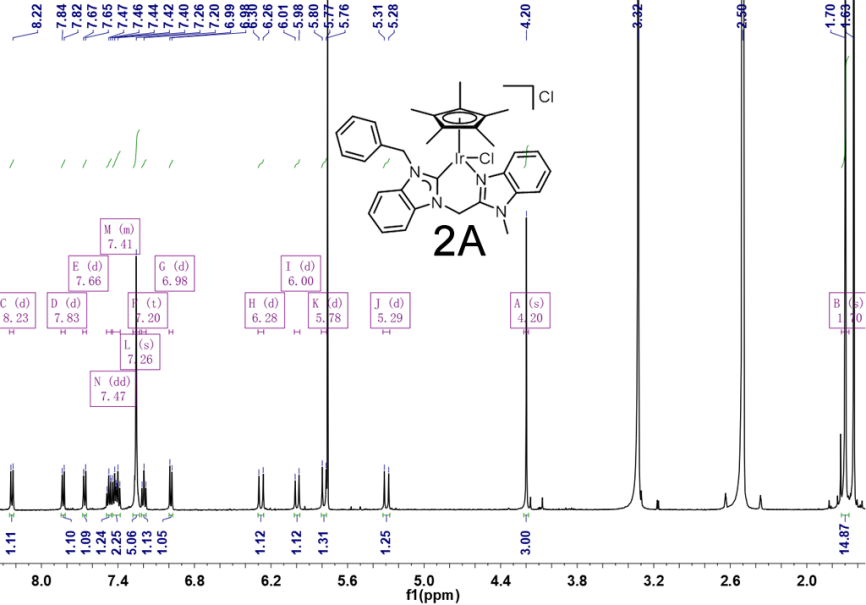


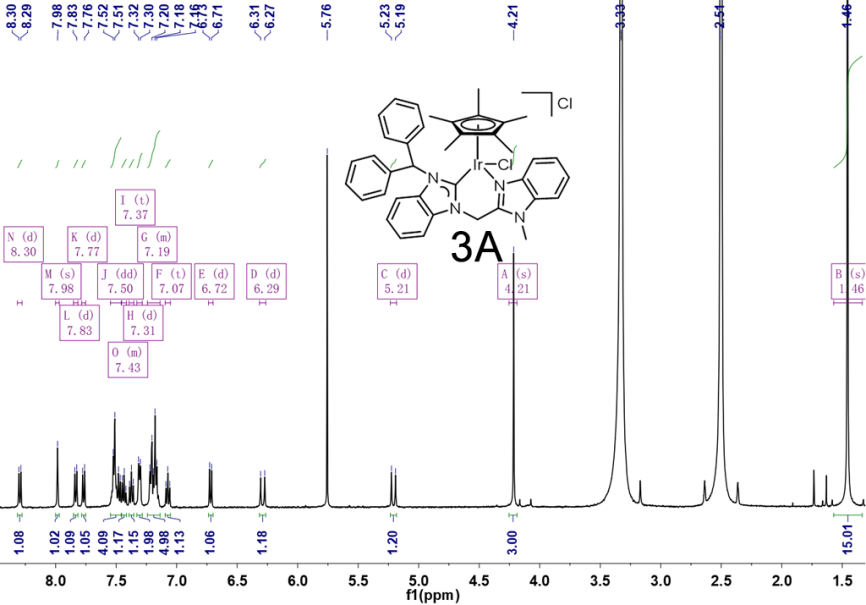


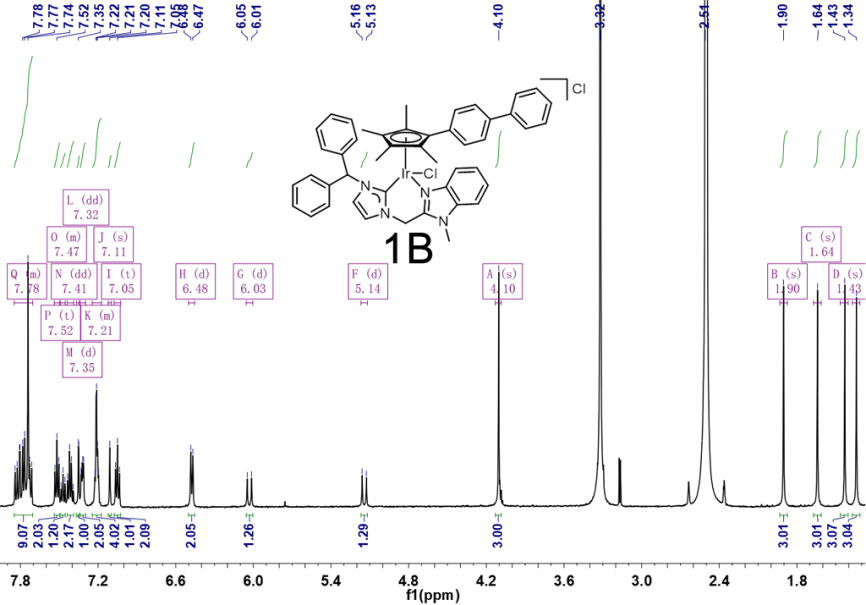


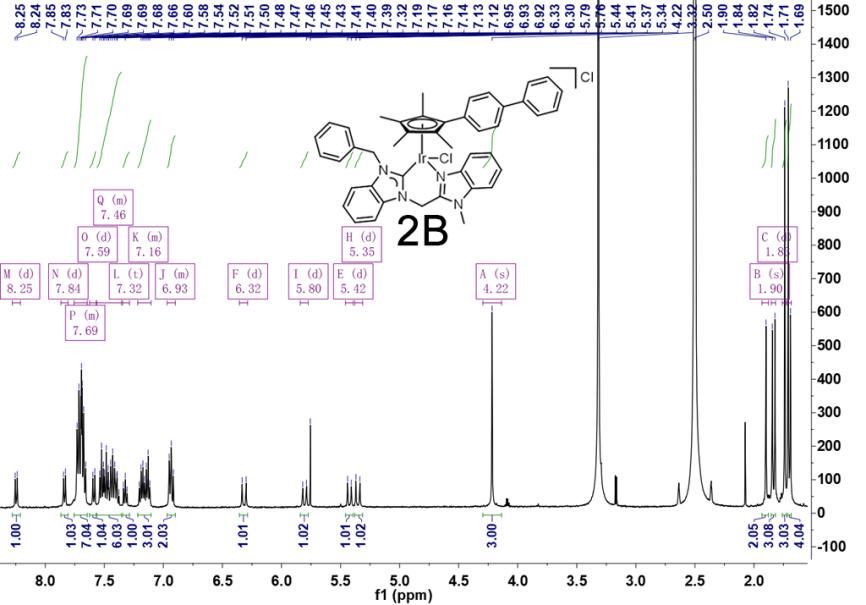


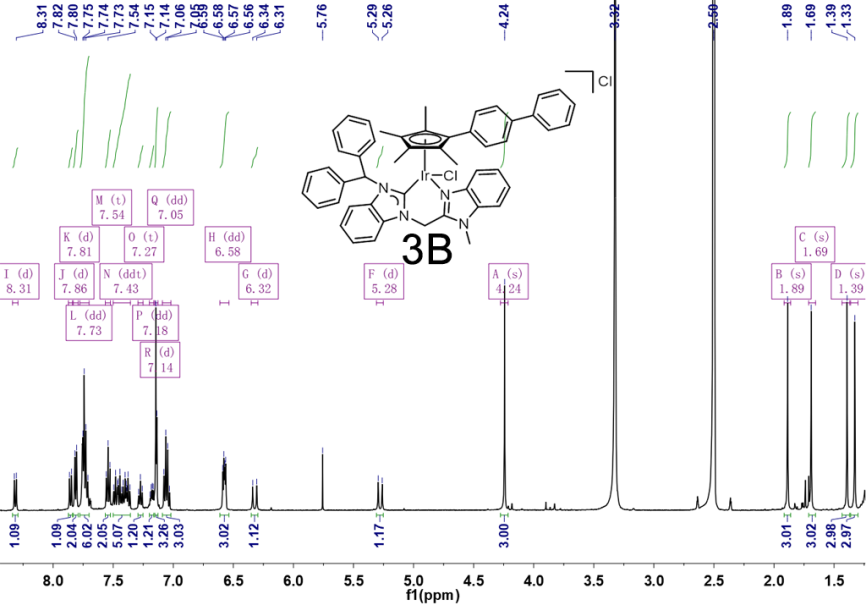


**Supplementary Figure 2** ^1^H NMR (500.13 MHz, DMSO) peak integrals of complex **1A-3B**.


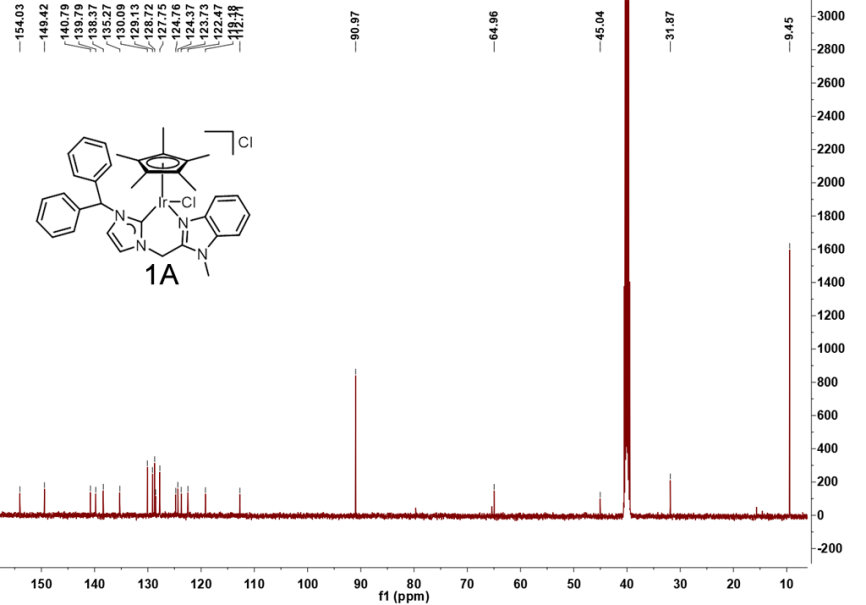


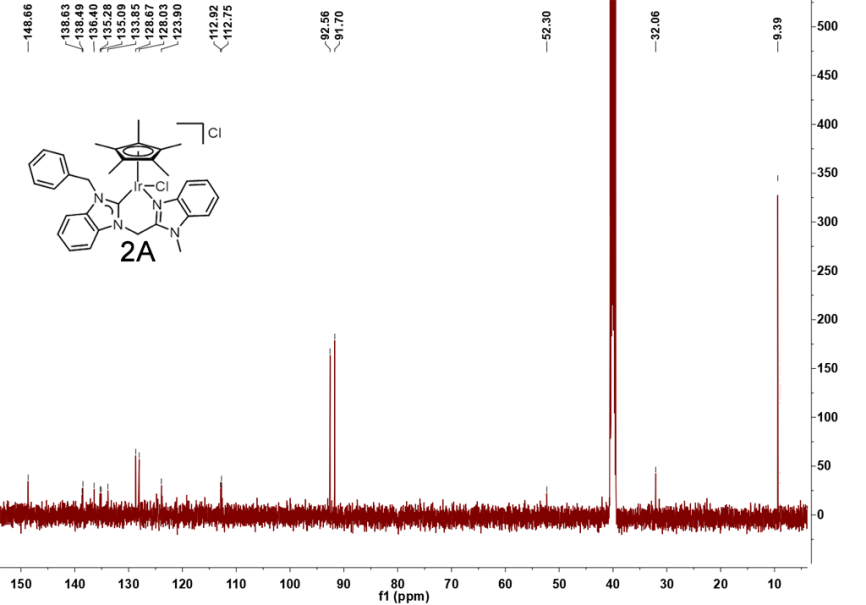


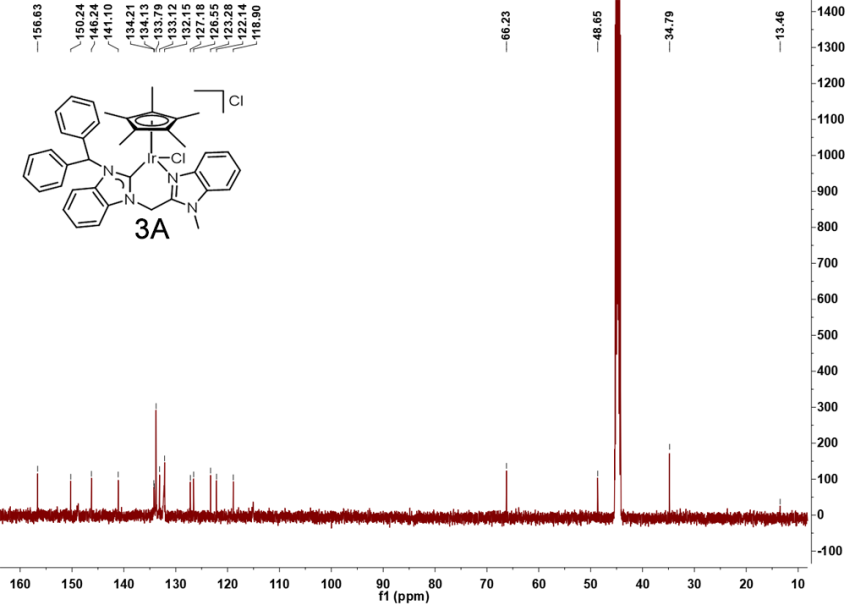


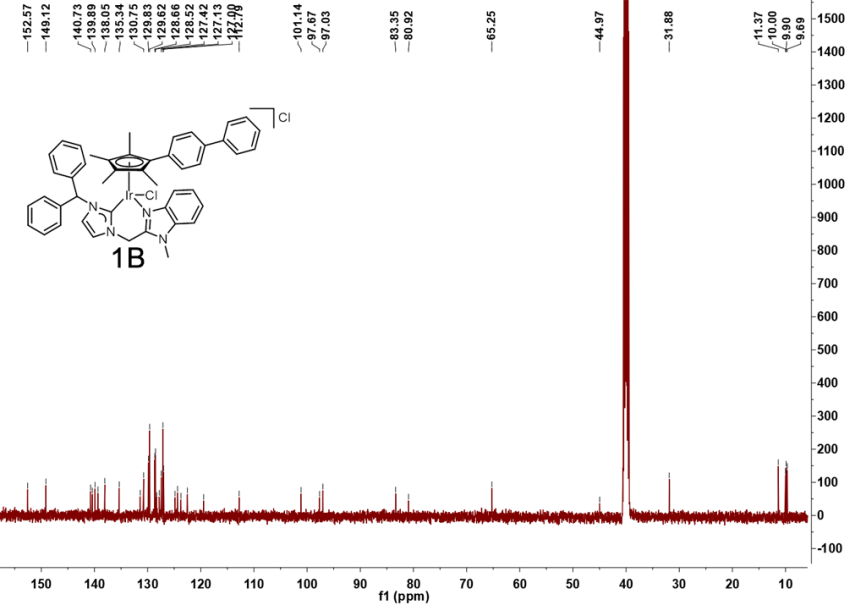


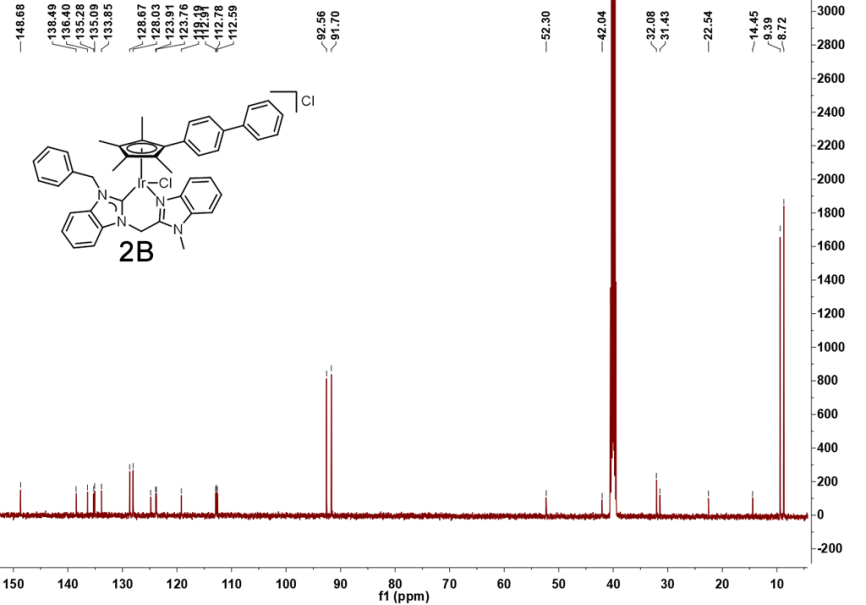


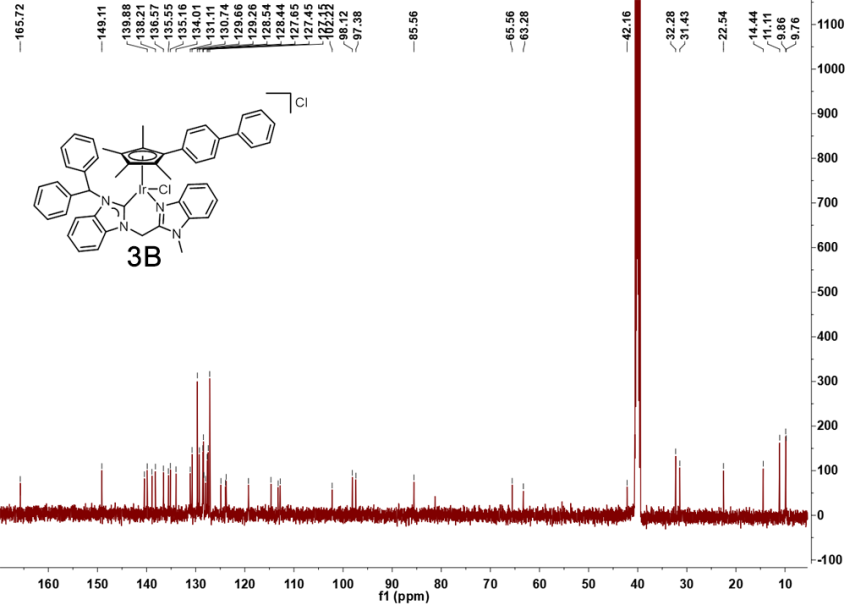


**Supporting Figure 3.** ^13^C NMR (126 MHz, DMSO) peak integrals of complexes **1A**-**3B**.


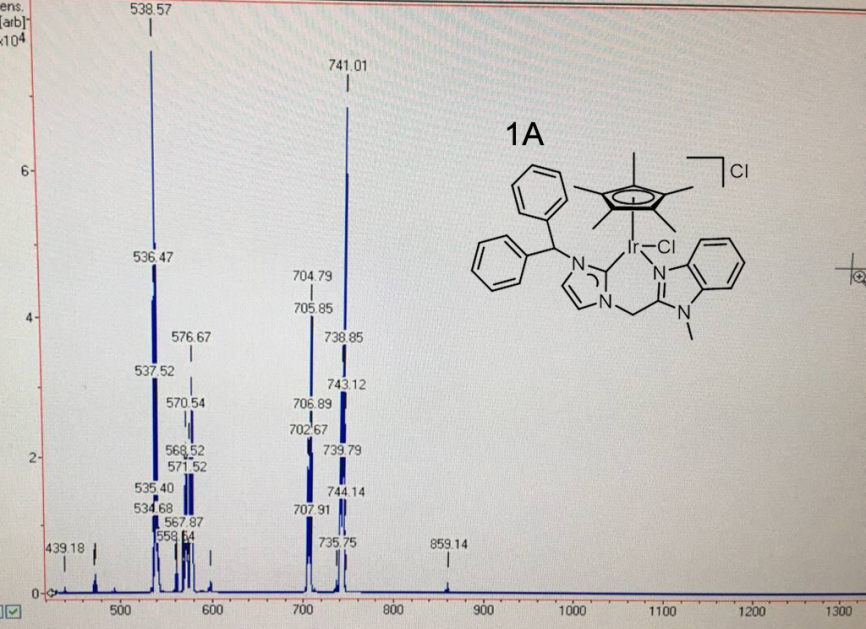


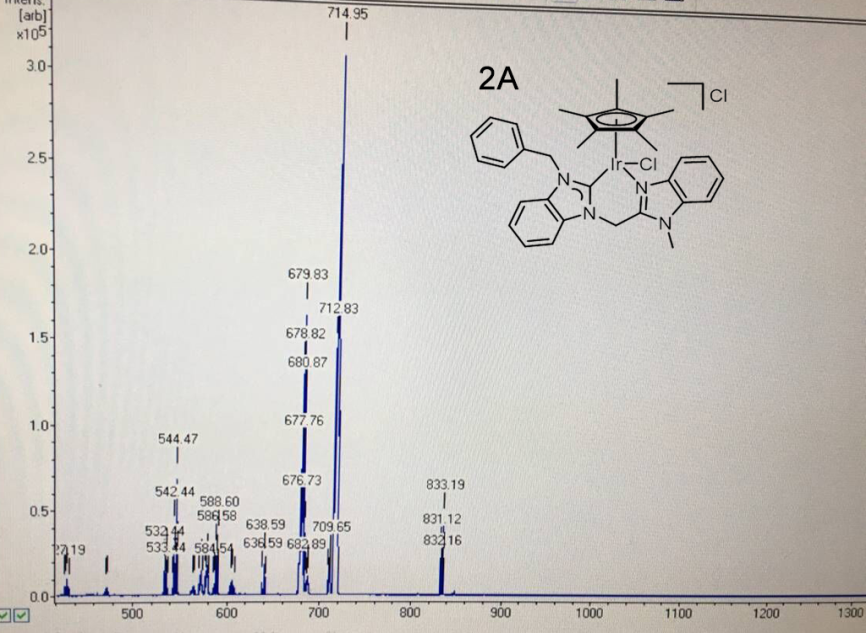


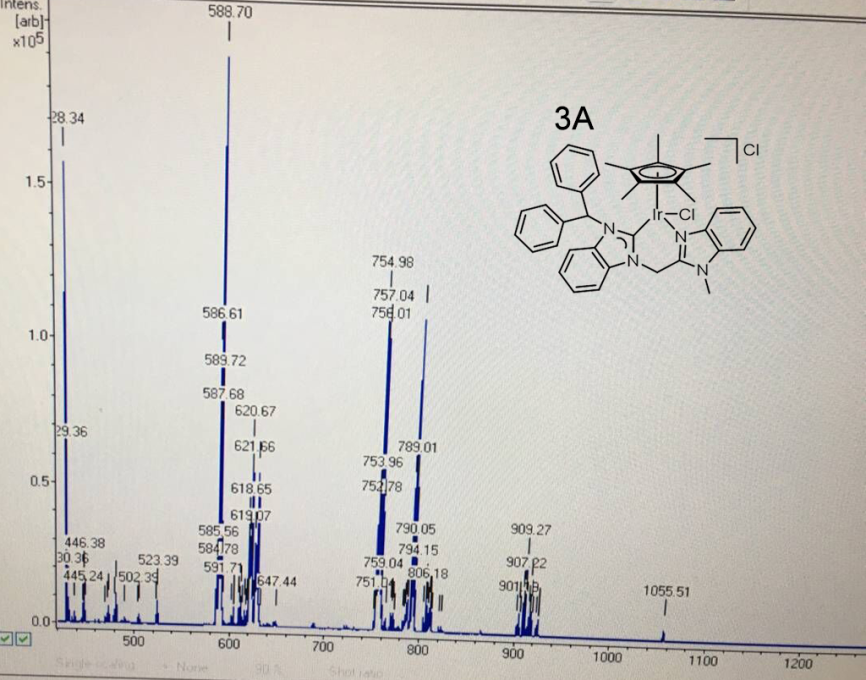


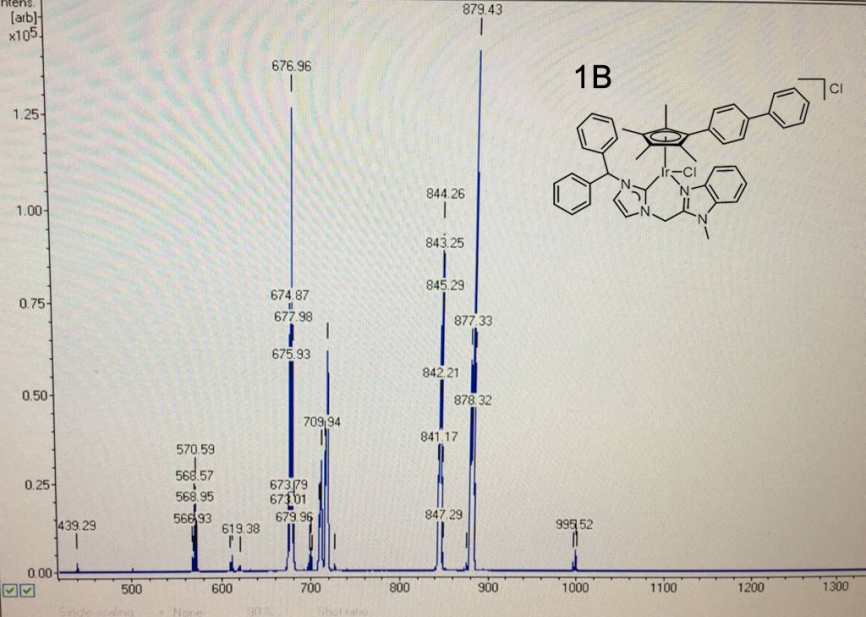


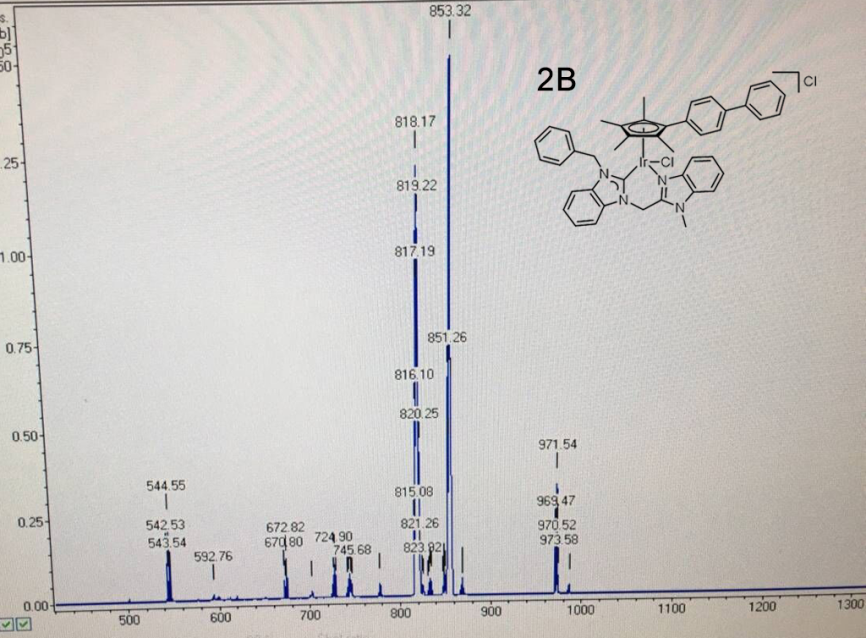


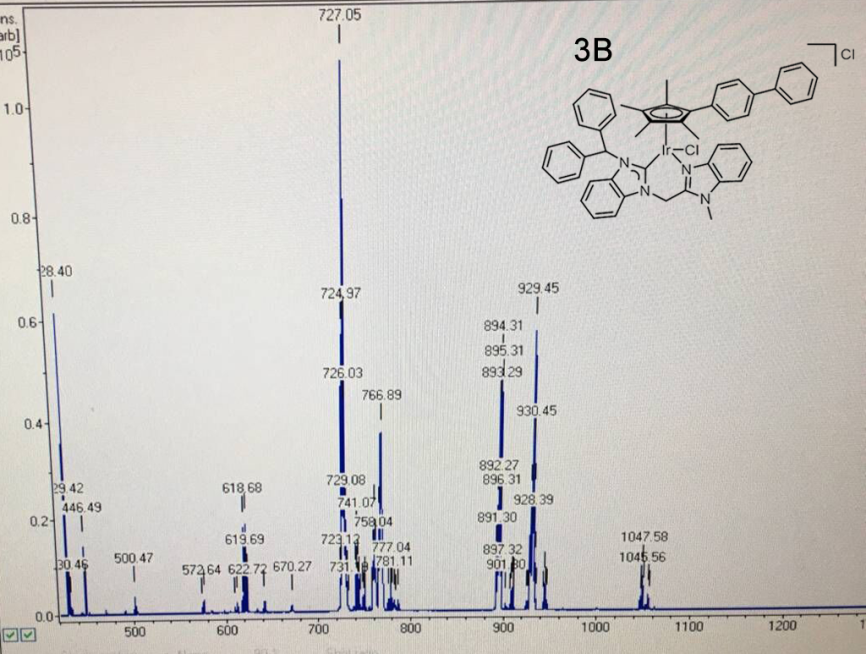


**Supporting Figure 4.** ESI-MS of complexes **1A**-**3B**.


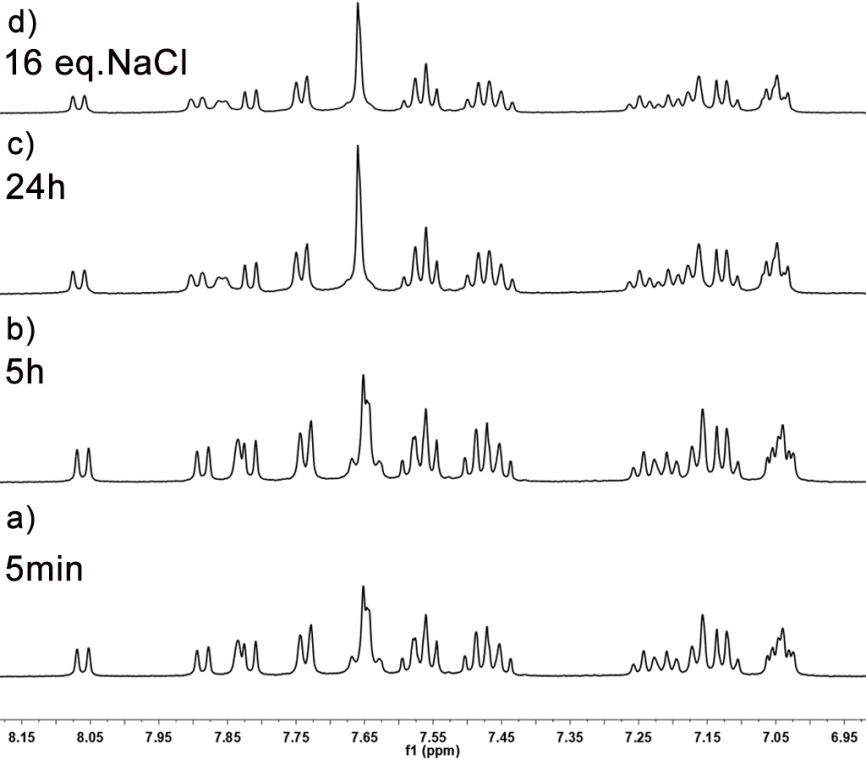


**Supporting Figure 5.** Low-field region of the ^1^H NMR spectra showing the hydrolysis of complex **3B** (1 mM) in 50% MeOD-*d*_4_/50% D_2_O (*v/v*) at 310 K. (a) after 5 min; (b) after 5 h ; (c) after 24 h; (d) add 16 eq. NaCl. No intereaction is observed.


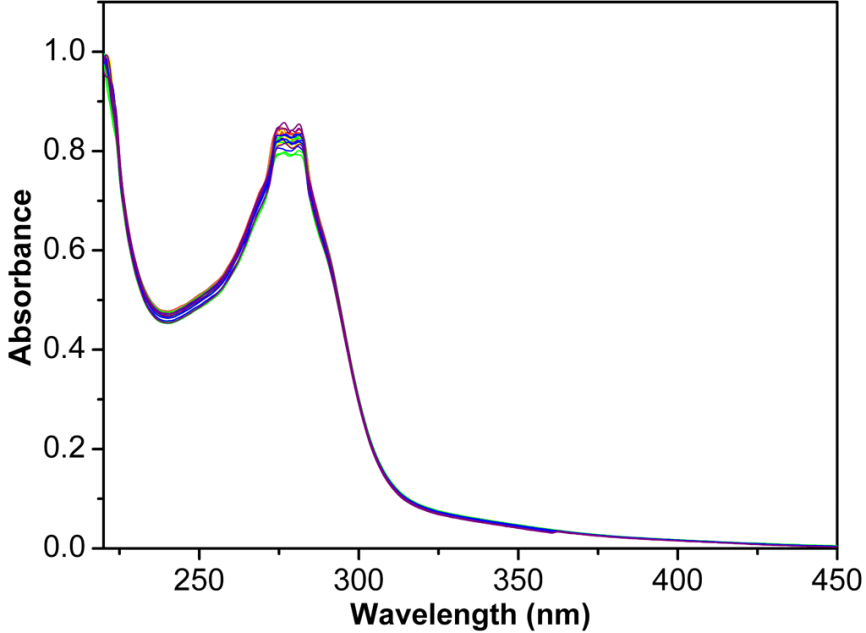


**Supporting Figure 6.** UV-Vis spectrum for a 50 µM solution of complex **3B** in 50% MeOH/50% H_2_O (*v/v*) recorded over a period of 24 h at 298


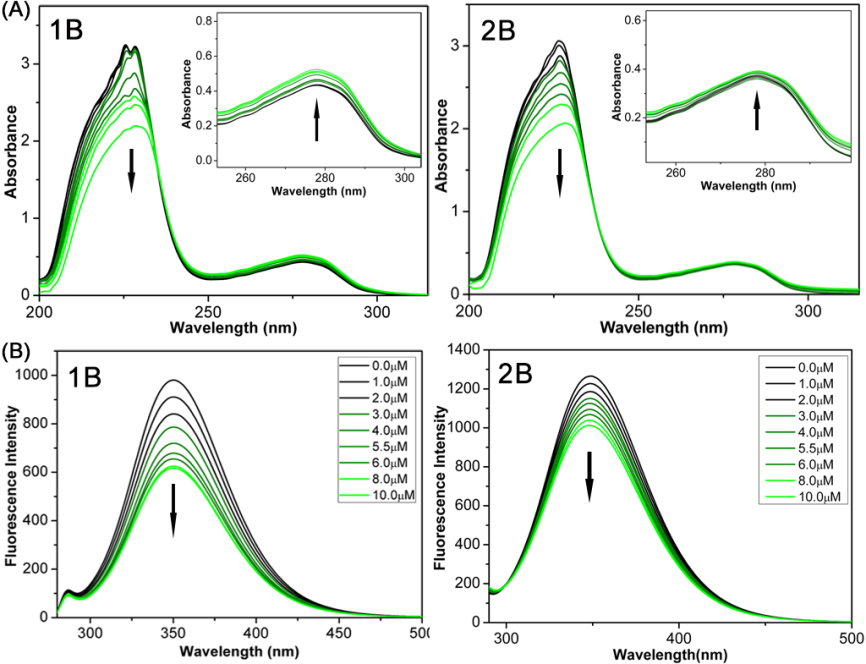


**Supporting Figure 7.** (A) UV-Vis spectroscopy of BSA in Tris-HCl/NaCI buffer solution (pH = 7.2) with the increase of complexes **1B** and **2B** (0-10 μM). Inset: Wavelength range: 250 nm-300 nm. (B) Fluorescence spectrum of BSA (*λ*_ex_ = 280 nm; *λ*_em_ = 343 nm) in the case where the concentration of complexes **1B** and **2B** was increased from 0 μM to 10 μM. Arrows: Direction of change in absorbance as the concentration of the complex increases.


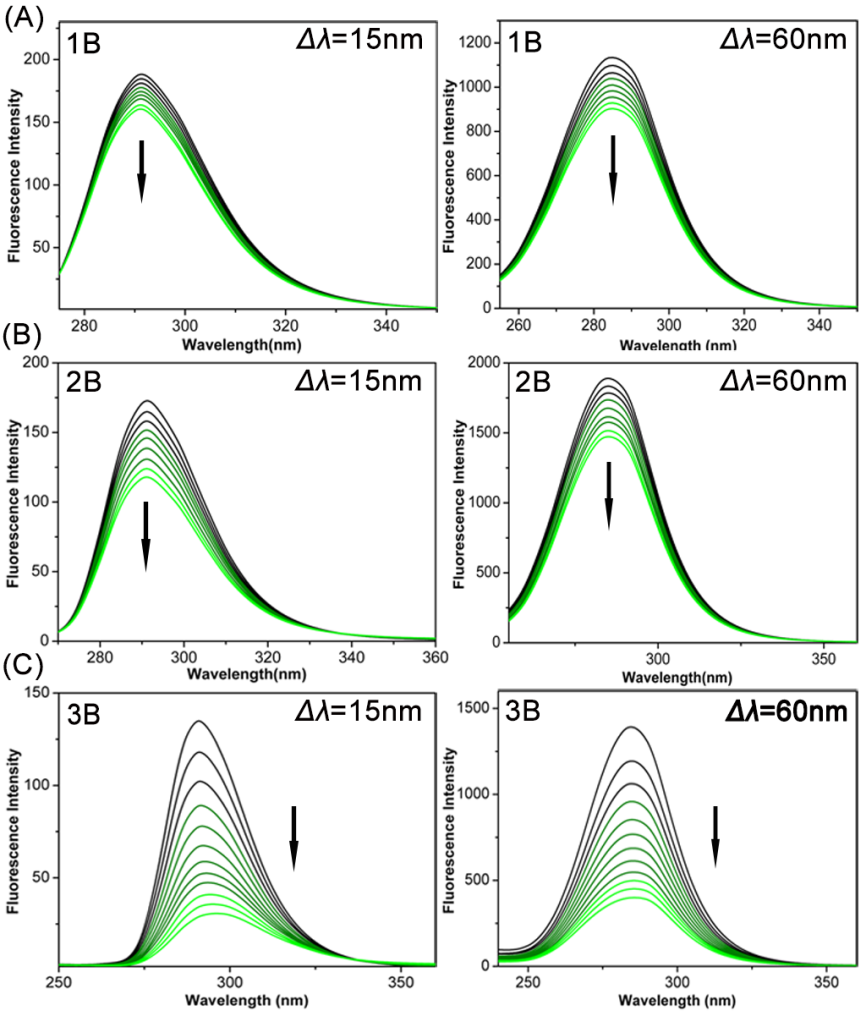


**Supporting Figure 8.** Synchronous spectra of BSA (10 μM) in the presence of increasing amounts of **1B** (A), **2B** (B) and **3B** (C) with a wavelength *Δλ* = 15 nm and *Δλ* = 60 nm.


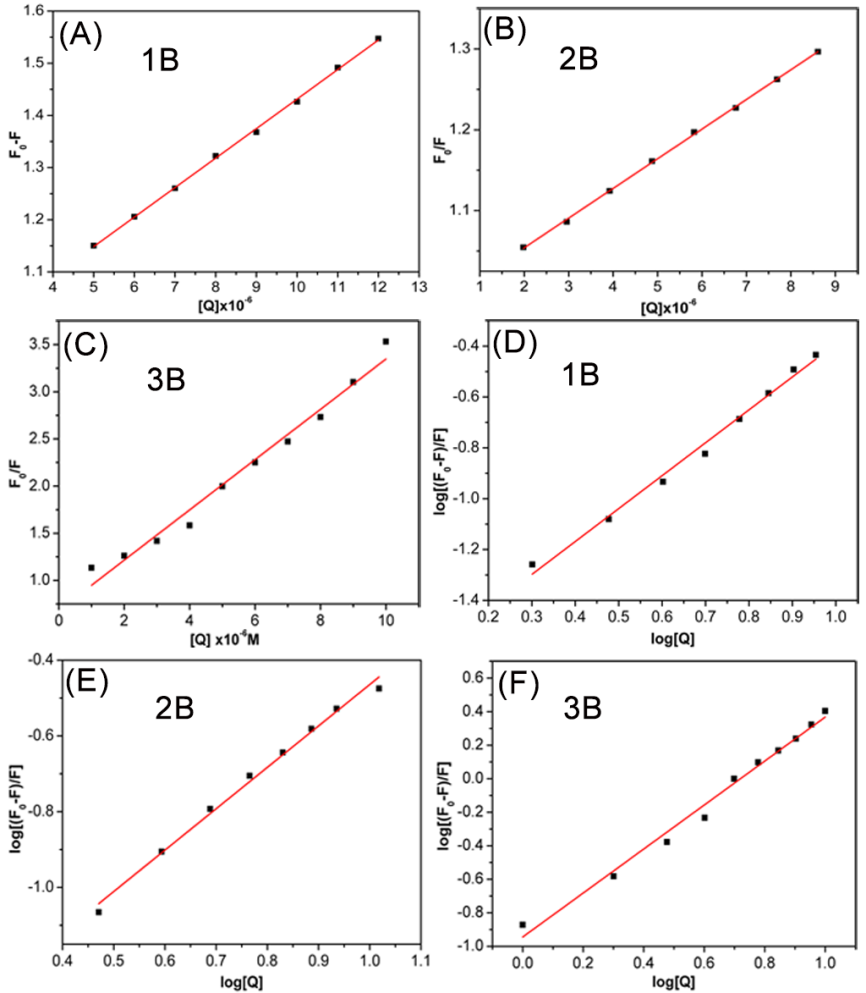


**Supporting Figure 9.** Stern-Volmer plots of F_0_/F against the concentration of complexes **1B**-**3B** (A)-(C) and plots of log[(F_0_-F)/F] vs. log[Q] for the interaction of BSA with complexes **1B-3B** (D)-(F).


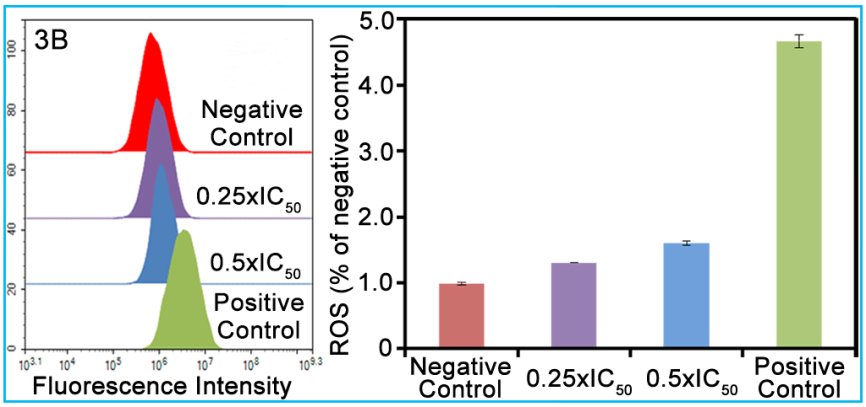


**Supporting Figure 10.** ROS levels were analyzed after A549 cells treated with complex **3B** (0.25 × IC_50_ and 0.5 × IC_50_) for 24 h. Data were referenced as average ±SD. of three repeated tests.


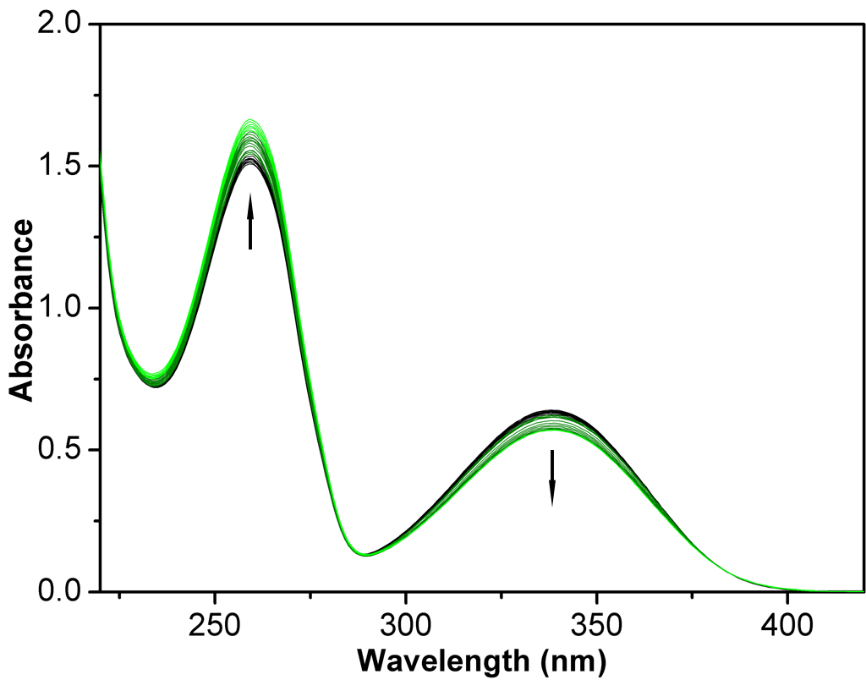


**Supporting Figure 11.** Reaction of complex **3B** (1.0 μM) and NADH (100 μM) in a 60% MeOH/40% H_2_O (*v/v*) mixed solution was monitored by UV-Vis at 298 K over 7 h. Arrows: The change trend of absorption over time.


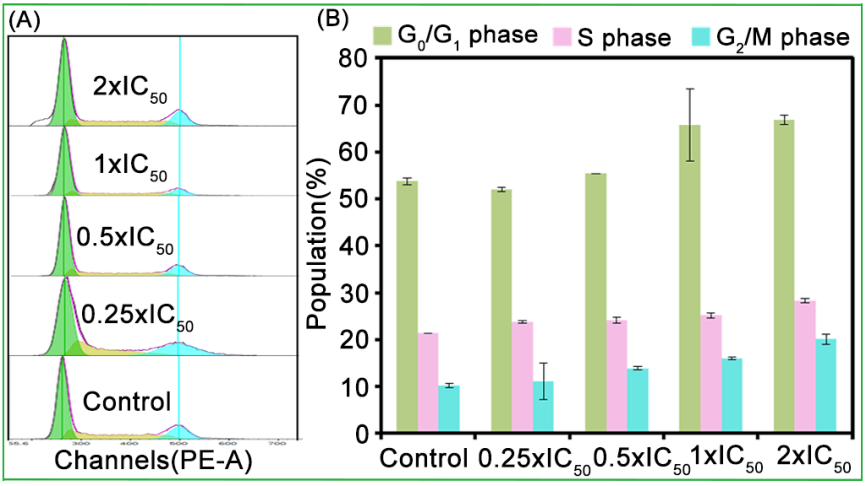


**Supporting Figure 12.** Cell cycle of A549 cells after exposed to complex **3B** at 310 K for 24 h. (A) The FL2 histograms of negative control group (untreated cells) and the concentration of complex **3B** were 0.25, 0.5, 1.0 and 2.0 × IC_50_, respectively. (B) The percentage of A549 cells treated with different concentrations of complex **3B** and negative control group at different stages of A549 cell cycle. Data were referenced as average ±SD of three repeated tests.

## 3 Supplementary Tables

**Supporting Table 1**. The table shows the data for the complex **1A**-**3B** ^1^H NMR spectra.

| **Complex** | **Hydrogen type** | **Chemical shift (ppm)** |
| --- | --- | --- |
| **1A-3A** | Cp*-C*H*_3_ | 1.44~1.70 |
| **1B-3B** | Cp^xbiph^-C*H*_3_ | 1.33~1.90 |
| **1A-3B** | N-C*H*_3_ | 4.08~4.24 |
| **1A-3B** | N-C*H*_2_C | 5.06~6.32 |
| **2A**, **2B** | N-C*H*_2_-Ar | 5.80~6.32 |
| **1A**, **1B**, **3A**, **3B** | N-C*H*-Ar_2_ | 7.49~7.98 |
| **1A, 1B** | Imidazole-*H* | 7.52~7.83 |
| **1A-3B** | Ar-*H* | 6.48~8.31 |

**Supporting Table 2**. The list shows the mass spectral data for complexes **1A-3B**.

| **Complex** | | **Mass spectrum peak** |
| --- | --- | --- |
| **1A** | calcd for [M-Cl]^+^: 741.23; Found: 741.01 | |
|  | calcd for [M-2Cl]^+^: 705.78; Found: 705.85 | |
| **2A** | calcd for [M-Cl]^+^: 715.22; Found: 714.95 | |
|  | calcd for [M-2Cl]^+^: 679.77; Found: 679.83 | |
| **3A** | calcd for [M-Cl]^+^: 789.55; Found: 790.05 | |
|  | calcd for [M-2Cl]^+^: 754.10; Found: 754.98 | |
| **1B** | calcd for [M-Cl]^+^: 879.28; Found: 879.43 | |
|  | calcd for [M-2Cl]^+^: 843.83; Found: 843.25 | |
| **2B** | calcd for [M-Cl]^+^: 853.26; Found: 853.32 | |
|  | calcd for [M-2Cl]^+^: 817.81; Found: 818.17 | |
| **3B** | calcd for [M-Cl]^+^: 929.24; Found: 929.45 | |
|  | calcd for [M-2Cl]^+^: 893.79; Found: 894.31 | |

**Supporting Table 3**. List of binding parameters for complexes **1B-3B** interaction with BSA.

| **Complex** | ***K_sv_* (10^4^M^-1^)** | ***K_q_* (10^12^ M^-1^s^-1^)** | ***K_b_* (10^4^** **M^-1^)** | **n** |
| --- | --- | --- | --- | --- |
| **1B** | 1.82 ± 2.1 | 1.82 | 1.07 | 0.96 |
| **2B** | 2.34 ± 0.6 | 2.34 | 1.11 | 1.12 |
| **3B** | 2.66 ± 0.2 | 2.66 | 1.13 | 1.31 |

**Supporting Table 4**. Flow cytometry analysis to determine the percentages of apoptotic cells, using Annexin V-FITC *vs* PI staining, after exposing A549 cells to complex **3B**.

|  | **Population (%)** | | | | |
| --- | --- | --- | --- | --- | --- |
| **Complex** | **Ir concentration** | **Viable** | **Early apoptosis** | **Late apoptosis** | **Non-viable** |
| **Control** | **——** | 93.6 ± 0.4 | 1.6 ±0.1 | 4.2 ± 0.1 | 0.1 ± 0.0 |
| **3B** | **0.5×IC_50_** | 92.5 ± 0.1 | 3.2 ± 0.1 | 4.5 ± 0.4 | 0.1 ± 0.0 |
|  | **1.0×IC_50_** | 81.5 ± 0.3 | 5.7 ± 0.1 | 12.5 ± 0.4 | 0.1 ± 0.0 |
|  | **2.0×IC_50_** | 2.0 ± 0.1 | 56.2 ± 0.5 | 41.4 ± 0.3 | 0.3 ± 0.0 |

**Supporting Table 5**. ROS induction in A549 cancer cells treated with complex **3B**.

| **Complex** | **Ir concentration** | **ROS (% of negative control)** |
| --- | --- | --- |
| **3B** | **Negative Control** | 0.9 ± 0.0 |
|  | **0.25×IC_50_** | 1.3 ± 0.1 |
|  | **0.50×IC_50_** | 1.6 ± 0.1 |
|  | **Positive Control** | 4.6 ± 0.1 |

**Supporting Table 6**. Cell cycle analysis carried out by flow cytometry using PI staining after exposing A549 cells to complex **3B**.

|  | **Population (%)** | | | |
| --- | --- | --- | --- | --- |
| **Complex** | **Ir concentration** | **G_0_/G_1_ phase** | **S phase** | **G_2_/M phase** |
| **Control** | **——** | 53.7 ± 0.6 | 21.3 ± 0.0 | 10.1 ± 0.5 |
| **3B** | **0.25×IC_50_** | 52.0 ± 0.4 | 23.8 ± 0.2 | 11.1 ± 3.8 |
|  | **0.5×IC_50_** | 55.3 ± 0.1 | 24.0 ± 0.6 | 13.8 ± 0.4 |
|  | **1.0×IC_50_** | 65.7 ± 7.7 | 25.1 ± 0.5 | 15.9 ± 0.2 |
|  | **2.0×IC_50_** | 66.8 ± 0.9 | 28.2 ± 0.4 | 20.1 ± 1.0 |

**Supporting Table 7**. **3B** induces mitochondrial membrane potential polarization in A549 cells.

|  | **Population (%)** | | |
| --- | --- | --- | --- |
| **Complex** | **Ir concentration** | **JC-1 Aggregates** | **JC-1 Monomers** |
| **Negative Control** | **——** | 92.7 ± 2.3 | 7.2 ± 0.3 |
| **Positive Control** | **——** | 24.6 ± 6.5 | 75.3 ± 8.8 |
| **3B** | **0.25×IC_50_** | 88.9 ± 0.1 | 11.0 ± 0.3 |
|  | **0.5×IC_50_** | 66.6 ± 0.2 | 33.3 ± 0.1 |
|  | **1.0×IC_50_** | 46.0 ± 1.3 | 53.9 ± 2.1 |
|  | **2.0×IC_50_** | 22.5 ± 0.1 | 77.4 ± 0.7 |

**Supporting Table 8**. Amount of **3B** distributed in cytosol, nucleus, nuclear chromatin and cytoskeleton fractions of A549 cells after 24 h exposure.

|  |  |  | **Ru ng/10^6^ cells** | | |
| --- | --- | --- | --- | --- | --- |
| **cytosol** |  |  | **nucleus** | **nuclear chromatin** | **cytoskeleton** |
| 0.51±0.12 | |  | 0.10±0.08 | 0.05±0.08 | 0.06±0.05 |

**
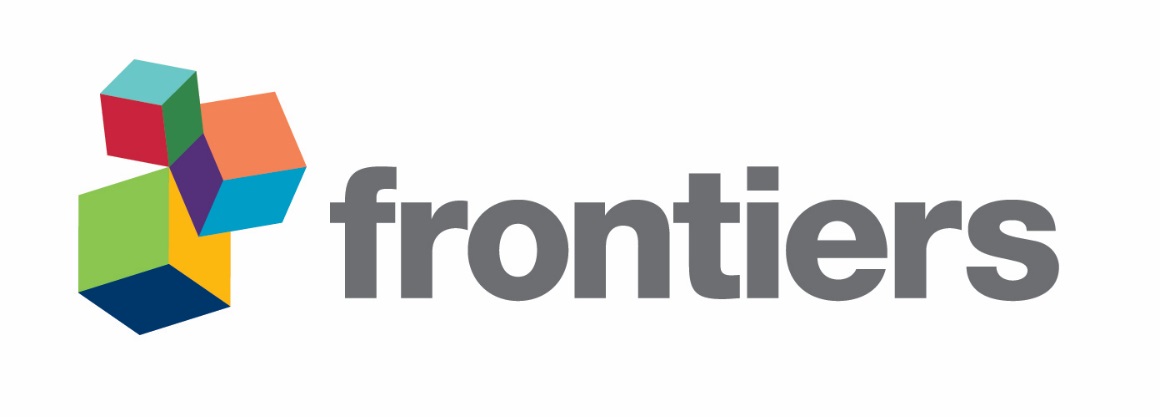
**
